# Supplementary material for: Sesquiterpenes and Monoterpenes from the Leaves and Stems of Illicium simonsii and Their Antibacterial Activity
Source: Molecules. 2022 Feb 8;27(3):1115. doi: 10.3390/molecules27031115 (PMC8838251; doi:10.3390/molecules27031115)
Supplement: Supplementary file 1 [file molecules-27-01115-s001.zip › molecules-1522882-supplementary.pdf]

Sesquiterpenes and Monoterpenes from the Leaves and Stems of *Illicium simonsii* and their Antibacterial Activity

Huijuan Li [1,2], Xinghui Song [1,2], Huiru Li [4], Lifei Zhu [2], Shengbo Cao [1,*] and Jifeng Liu [3,*]

[1] State Key Laboratory of Agricultural Microbiology, Huazhong Agricultural University, Wuhan, Hubei, 430070, P.R. China; sbcao@mail.hzau.edu.cn
[2] School of Veterinary Medicine, Henan University of Animal Husbandry and Economy, Zhengzhou, 450046, P.R. China; lihj2022@126.com
[3] School of Pharmaceutical Science, Zhengzhou University, Ke Xue Da Dao 100, Zhengzhou 450001, P.R. China; liujf2009y@126.com
[4] Eastern China Conservation Centre for Wild Endangered Plant Resources, Shanghai Chenshan Botanical Garden, Shanghai 201602, P.R. China
* Correspondence: sbcao@mail.hzau.edu.cn (S. C.); liujf2009y@126.com (J.L.)

Supporting information

Content

**Page S2-S13**	^{1}H NMR, ^{13}C NMR, DEPT-90, DEPT-135, HSQC, HMBC, COSY, ROESY, CD, HRESI, IR (KBr) and UV spectrum of compound **1**
**Page S14-S25**	^{1}H NMR, ^{13}C NMR, DEPT-90, DEPT-135, HSQC, HMBC, COSY, ROESY, CD, HRESI, IR (KBr) and UV spectrum of compound **3**
**Page S26-S36**	^{1}H NMR, ^{13}C NMR, DEPT-90, DEPT-135, HSQC, HMBC, COSY, ROESY, CD, HRESI, IR (KBr) and UV spectrum of compound **5**
**Page S37-S39**	Spectroscopic data of compound **2, 4** and **6-11**
**Page S40:**	K-B disk diffusion test against *S. aureus*, *B. subtilis* and *E. faecalis.*

The ^{1}H NMR spectrum of compound **1**

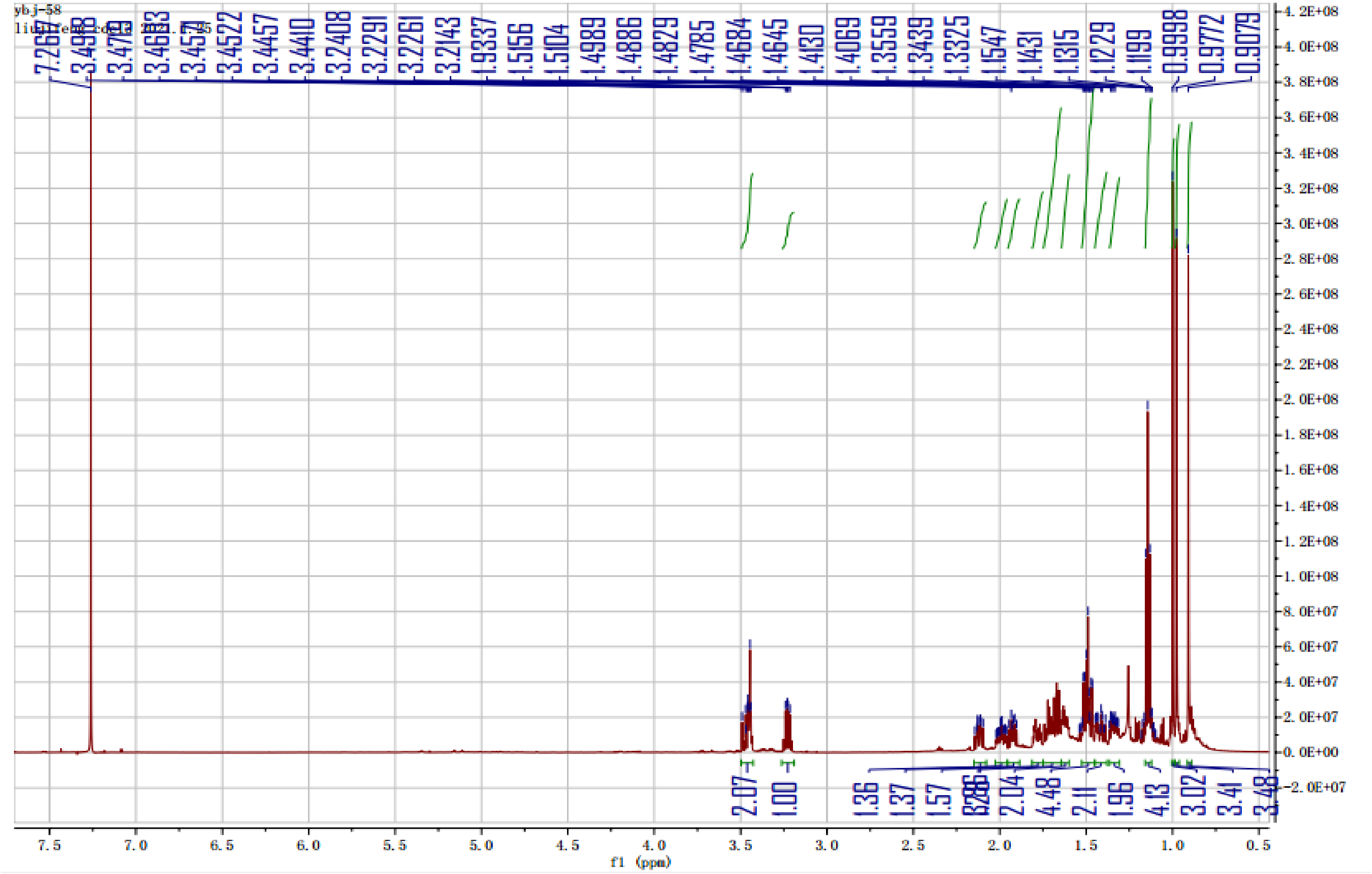

The ^{1}H NMR spectrum of compound **1**

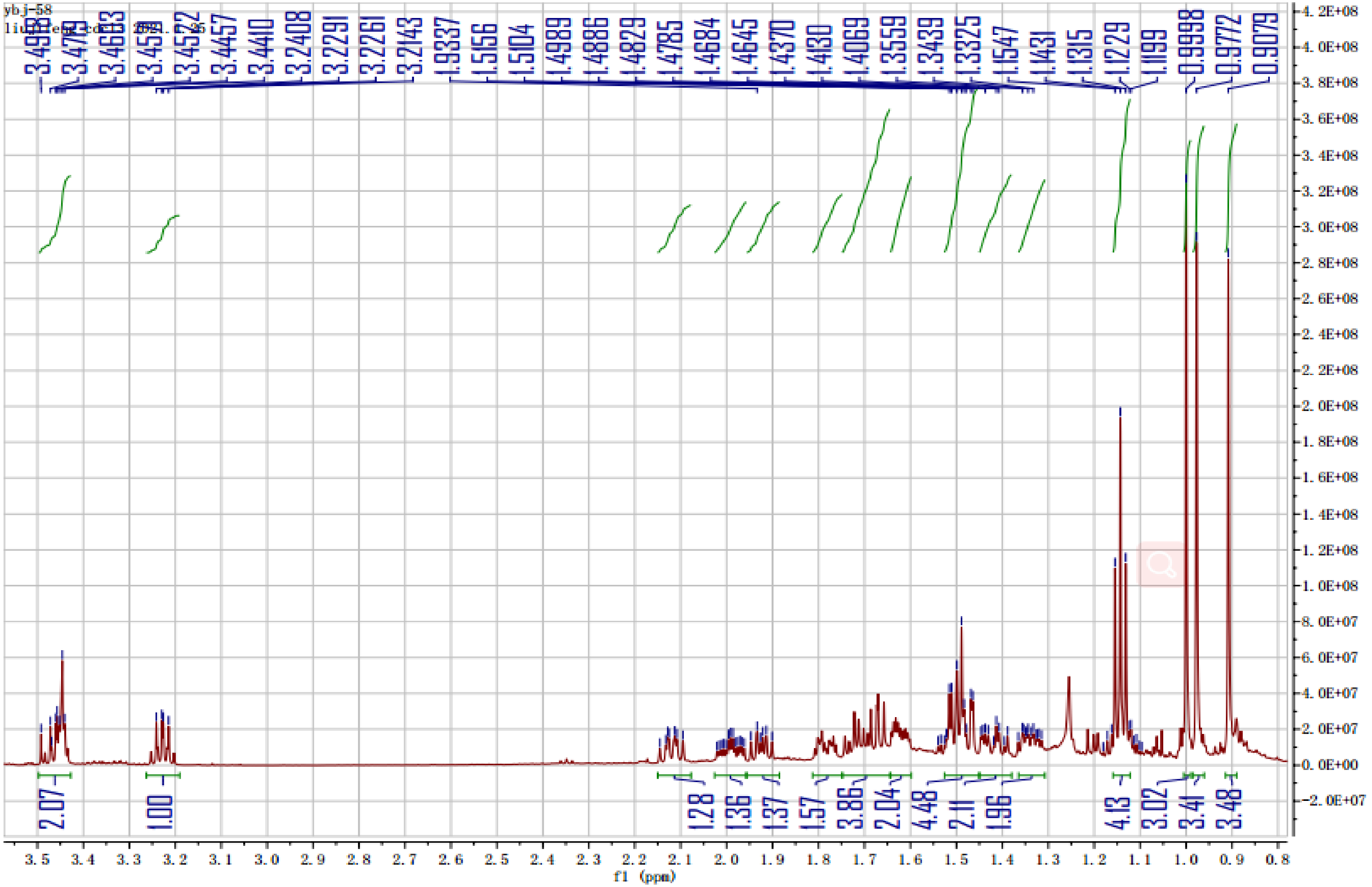

The ^{13}C NMR of compound **1**

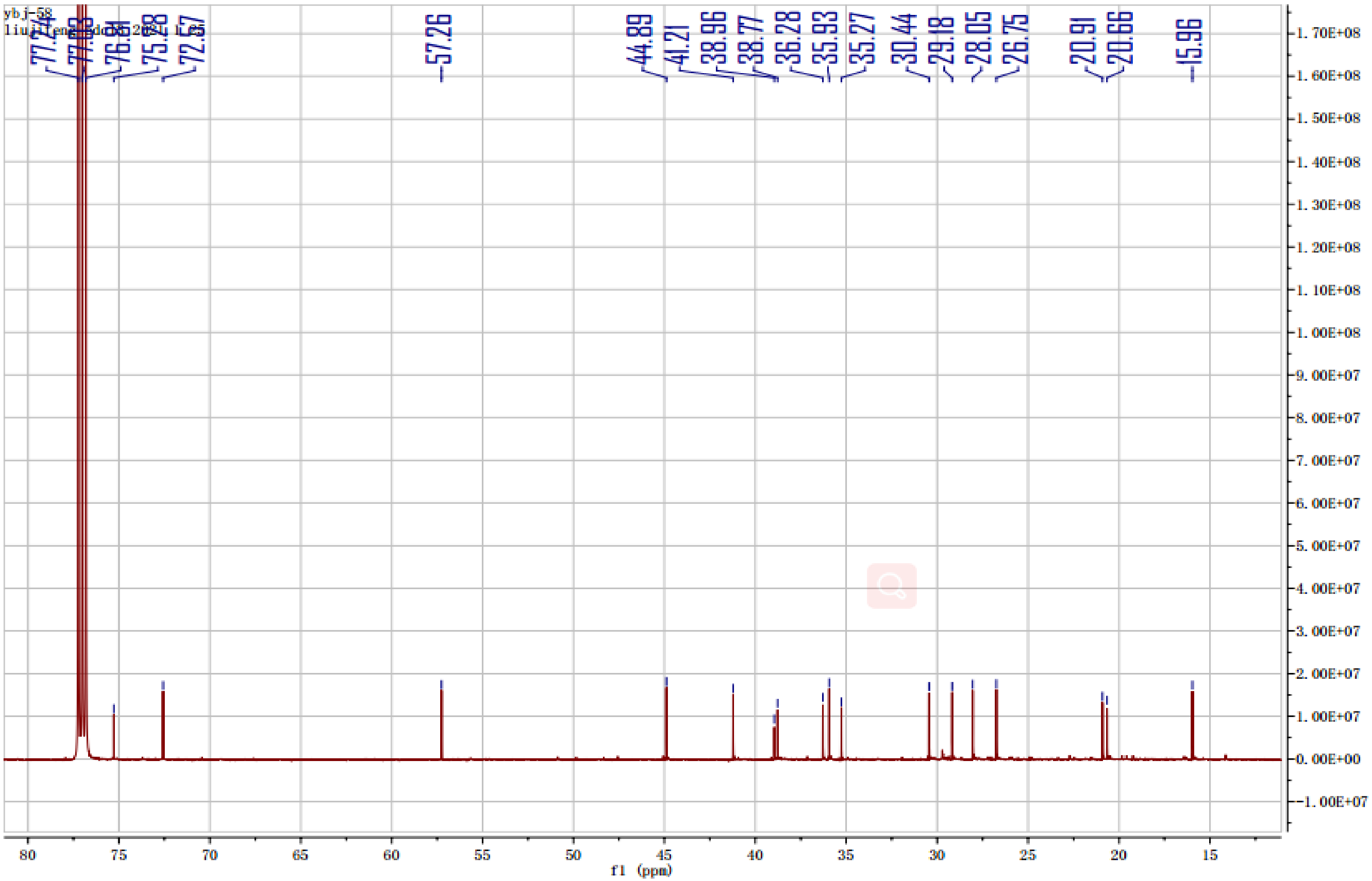

The DEPT-135 spectrum of compound **1**

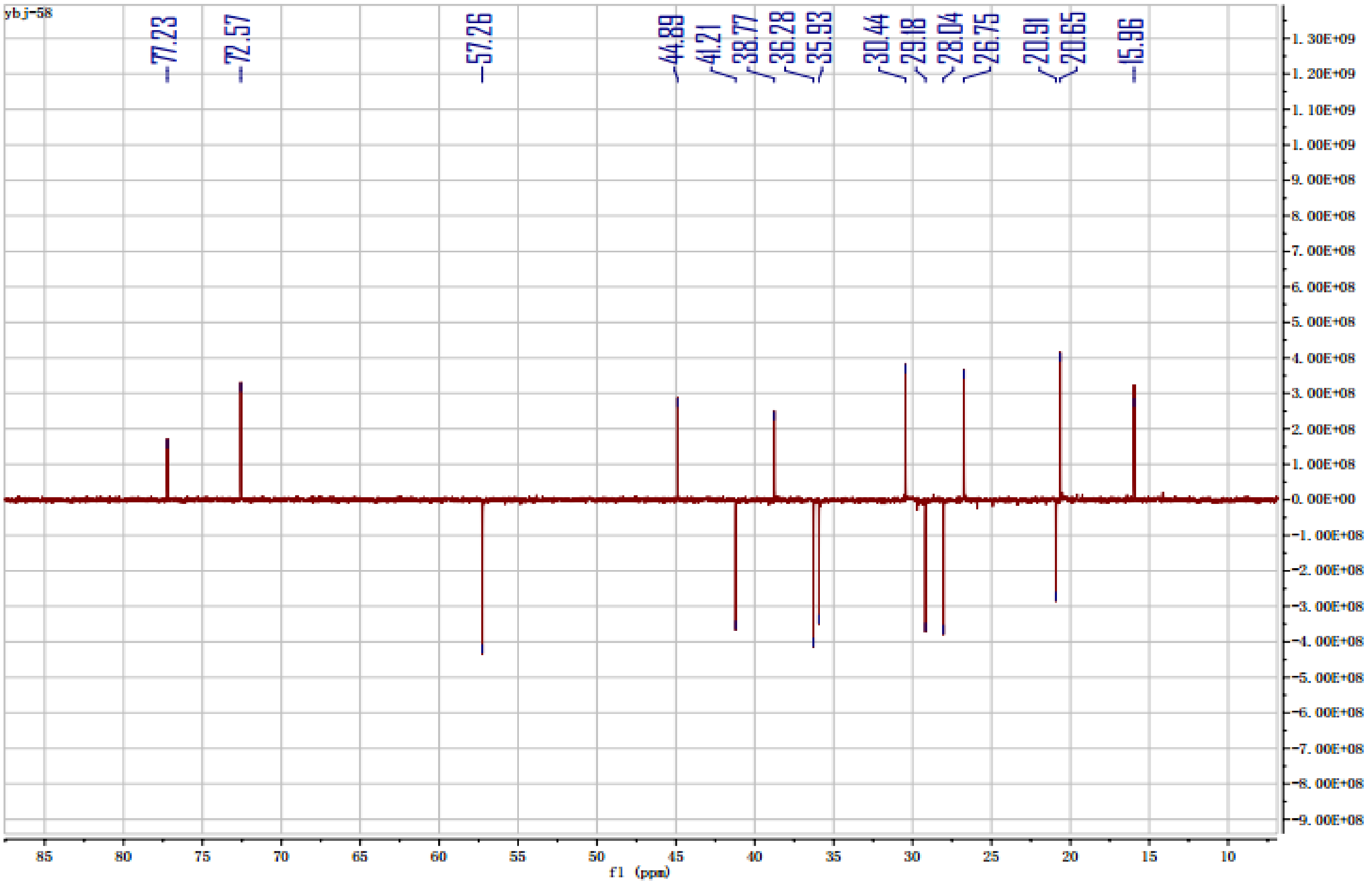

The DEPT-90 spectrum of compound **1**

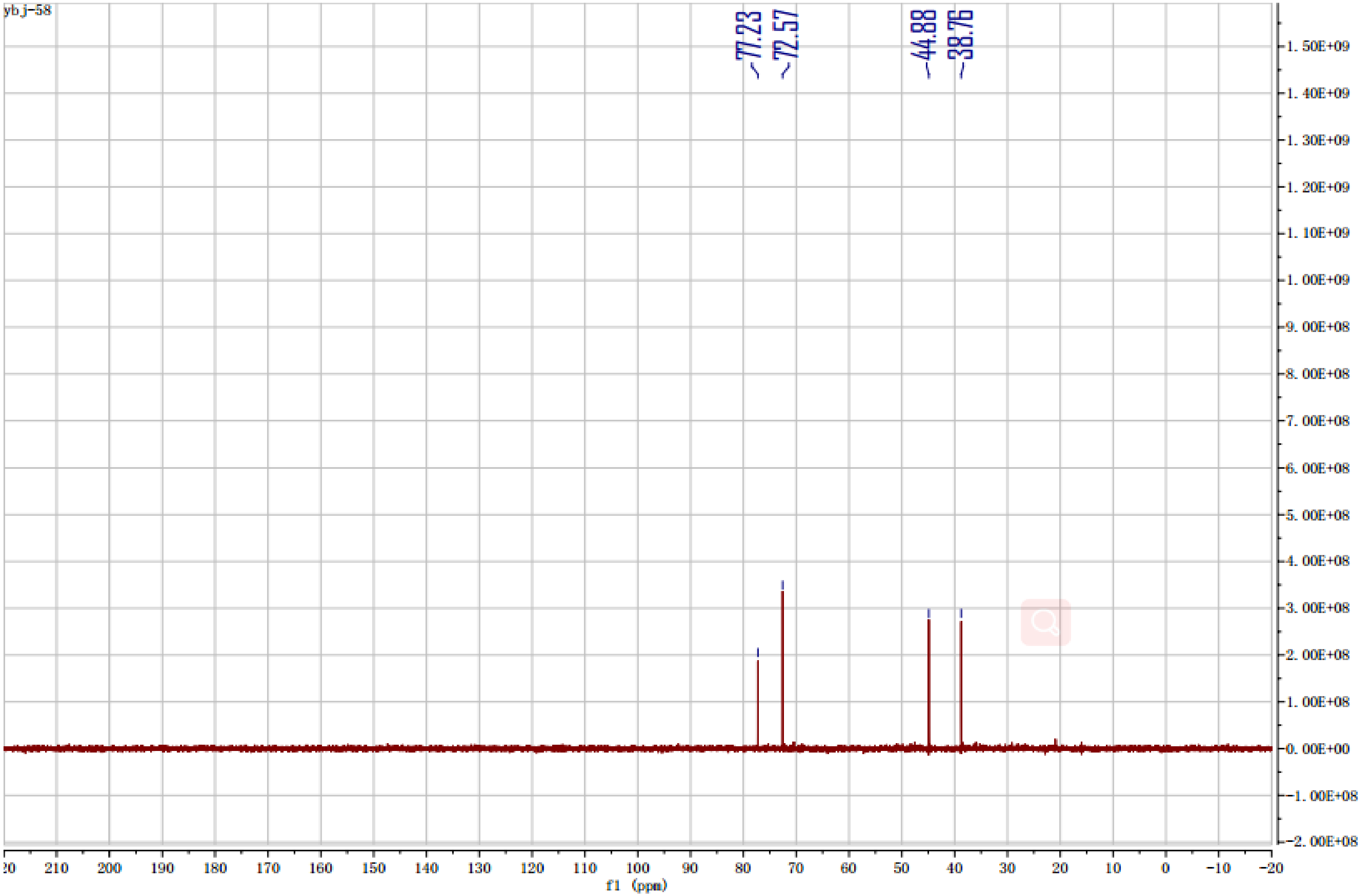

The HSQC spectrum of compound **1**

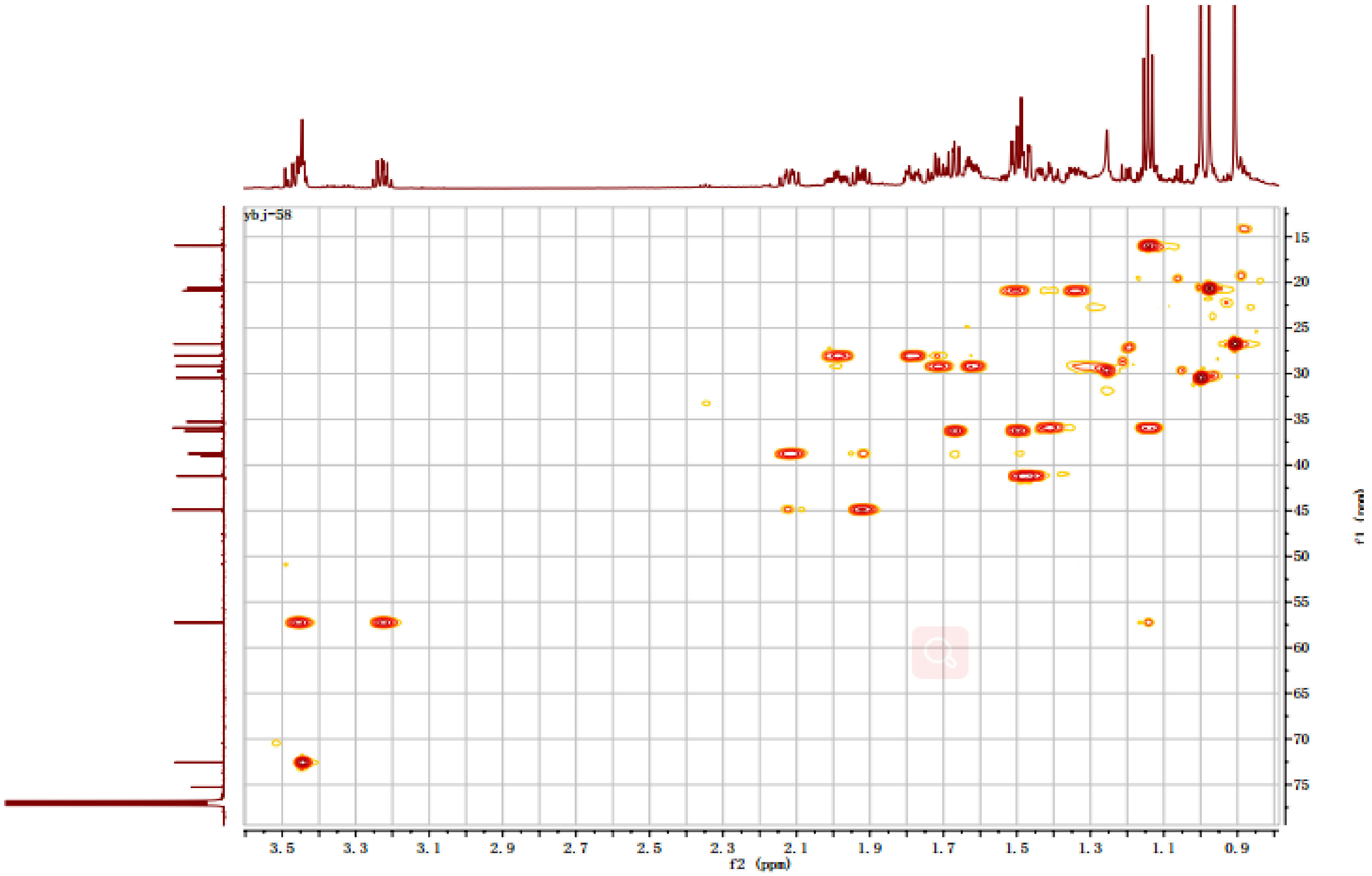

The HMBC spectrum of compound **1**

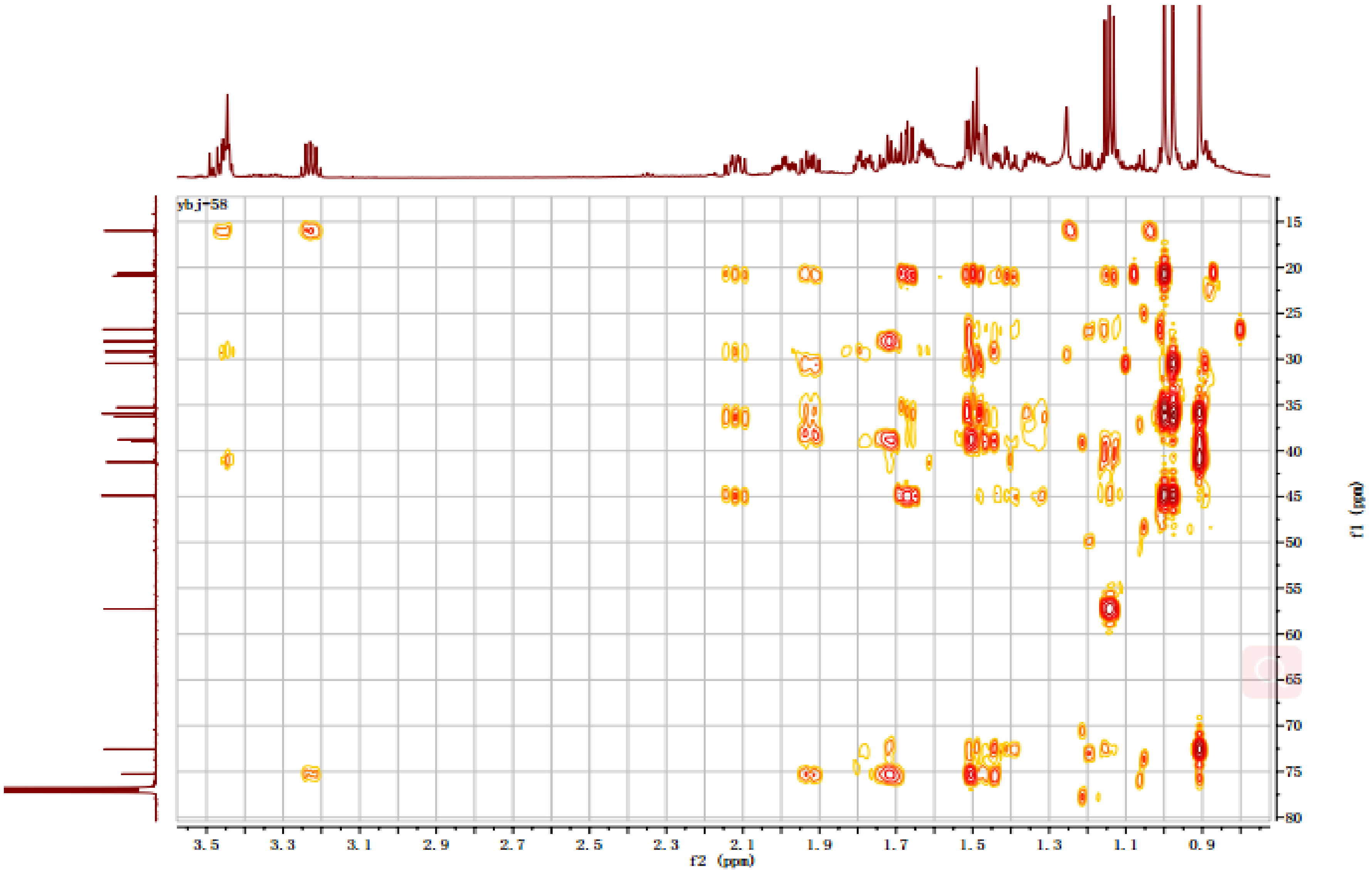

The COSY spectrum of compound **1**

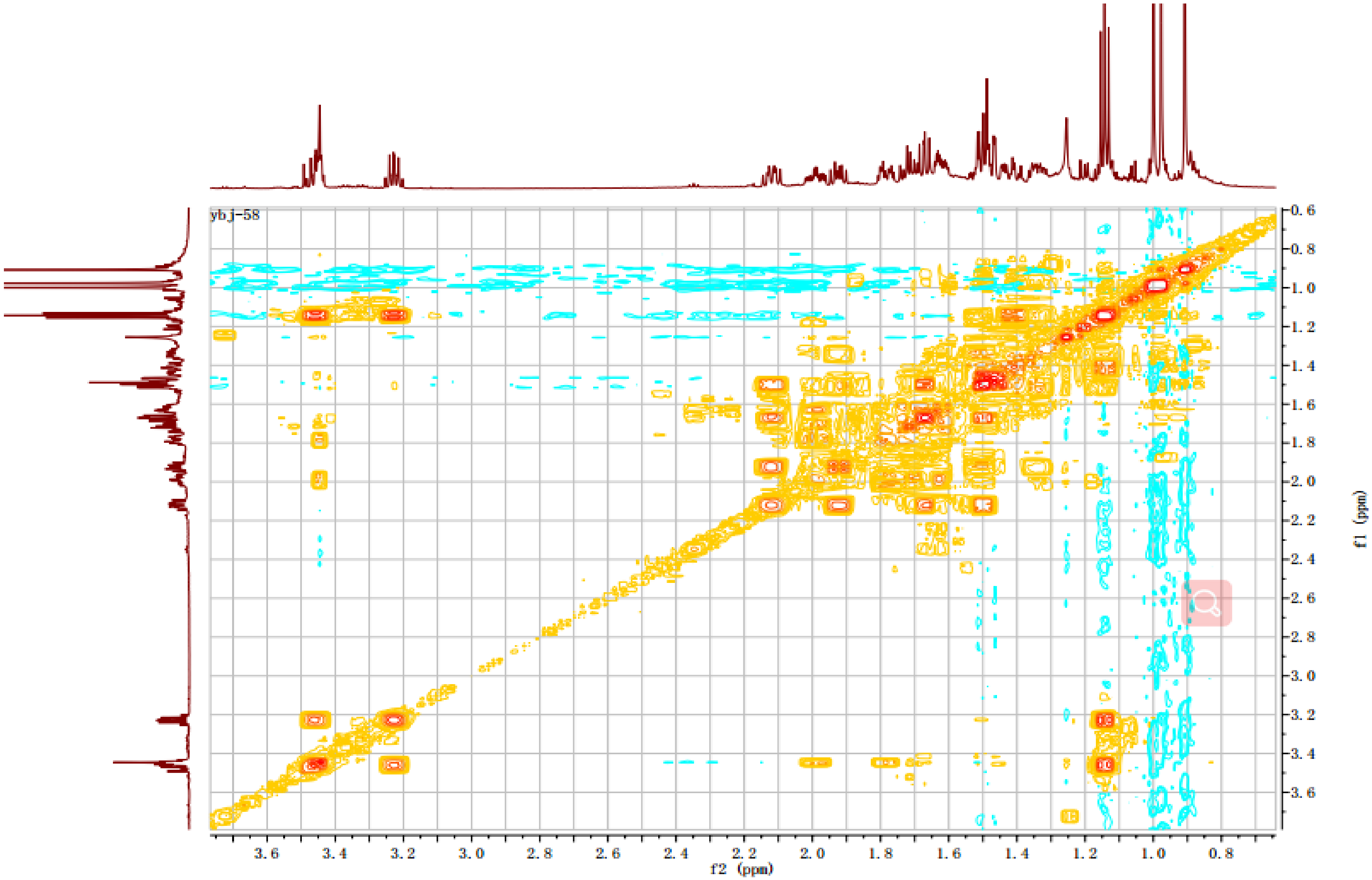

The ROESY spectrum of compound **1**

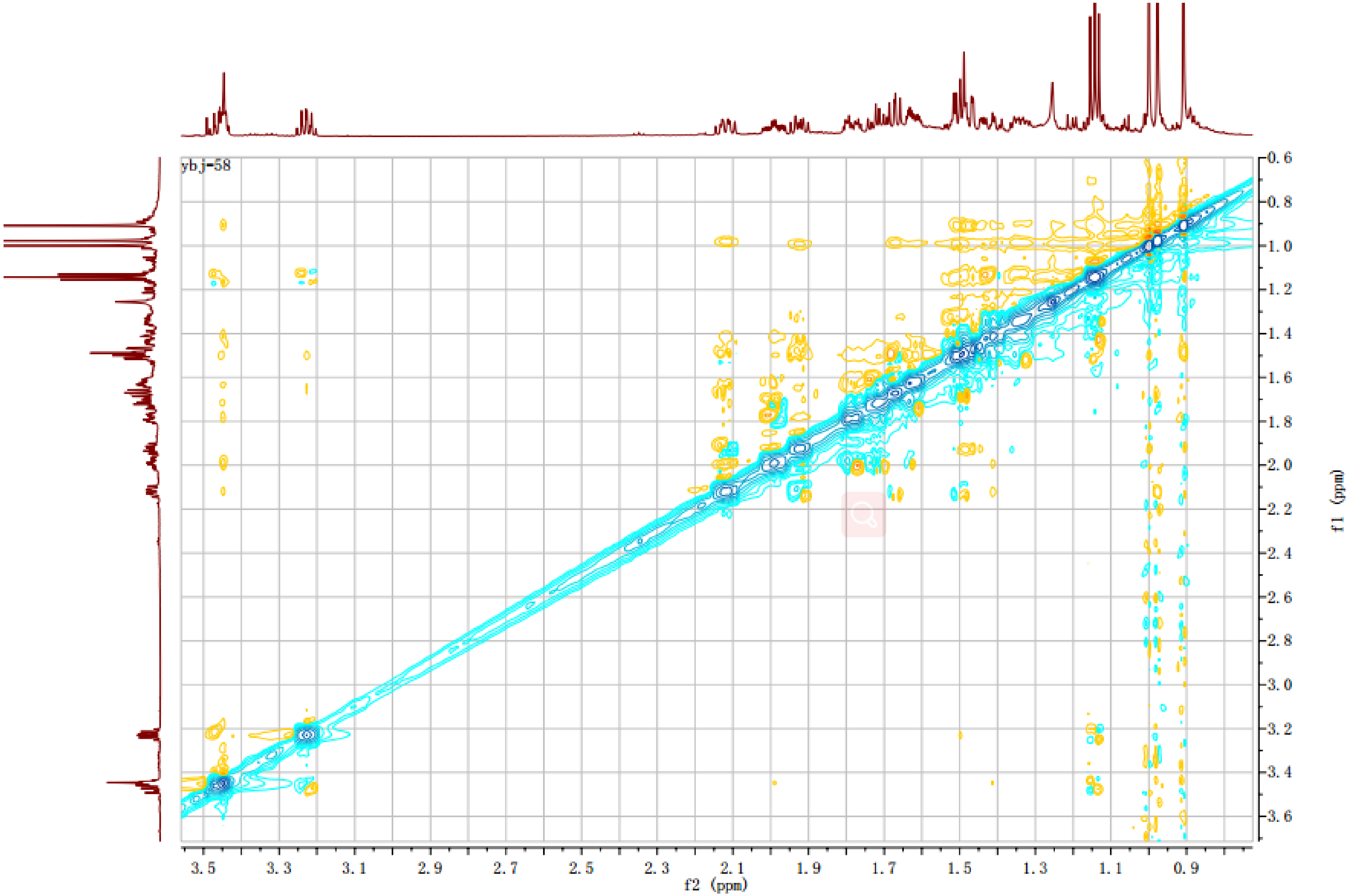

The HR-ESI-MS of compound **1**

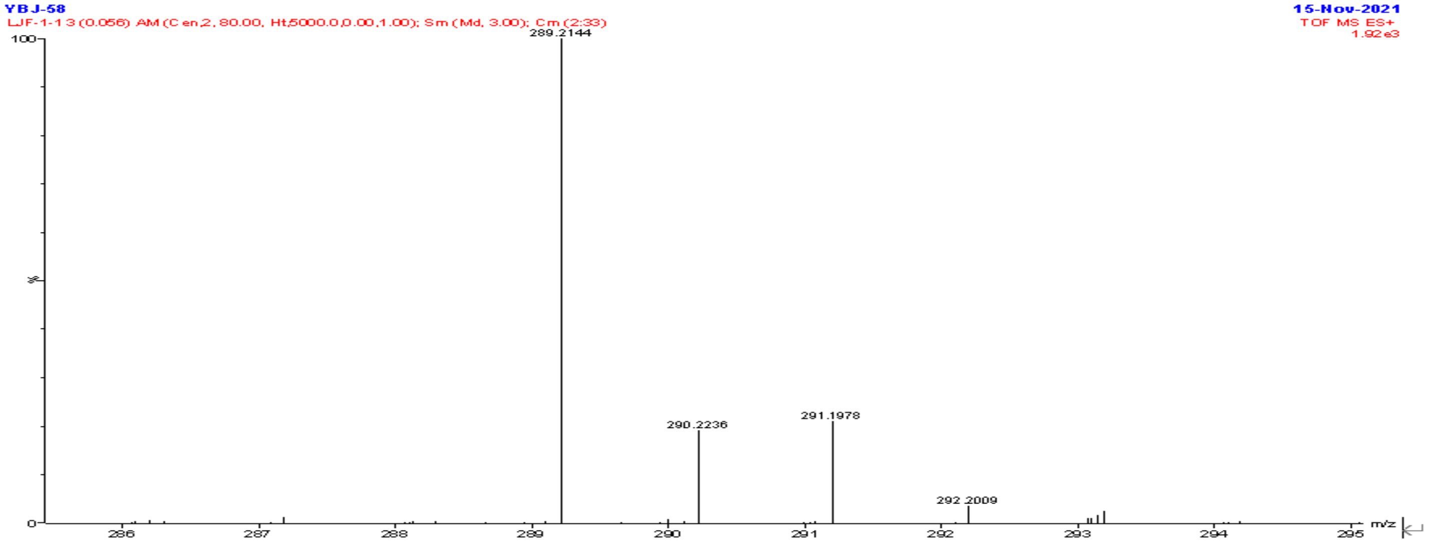

Elemental Composition Report

Single Mass Analysis

Tolerance = 50.0 PPM / DBE: min = -1.5, max = 50.0

Selected filters: None

Monoisotopic Mass, Even Electron Ions

92 formula(e) evaluated with 3 results within limits (all results (up to 1000) for each mass)

Elements Used:

C: 0-500 H: 0-1000 O: 0-20 Na: 0-1

Minimum:				-1.5		
Maximum:		10.0	50.0	50.0		
Mass	Calc. Mass	mDa	PPM	DBE	i-FIT	Formula
289.2144	289.2168	-2.4	-8.3	5.5	167.1	C19 H29 O2
	289.2144	0.0	0.0	2.5	169.8	C17 H30 O2 Na
	289.2015	12.9	44.6	1.5	175.6	C15 H29 O5

The IR (KBr) of compound **1**

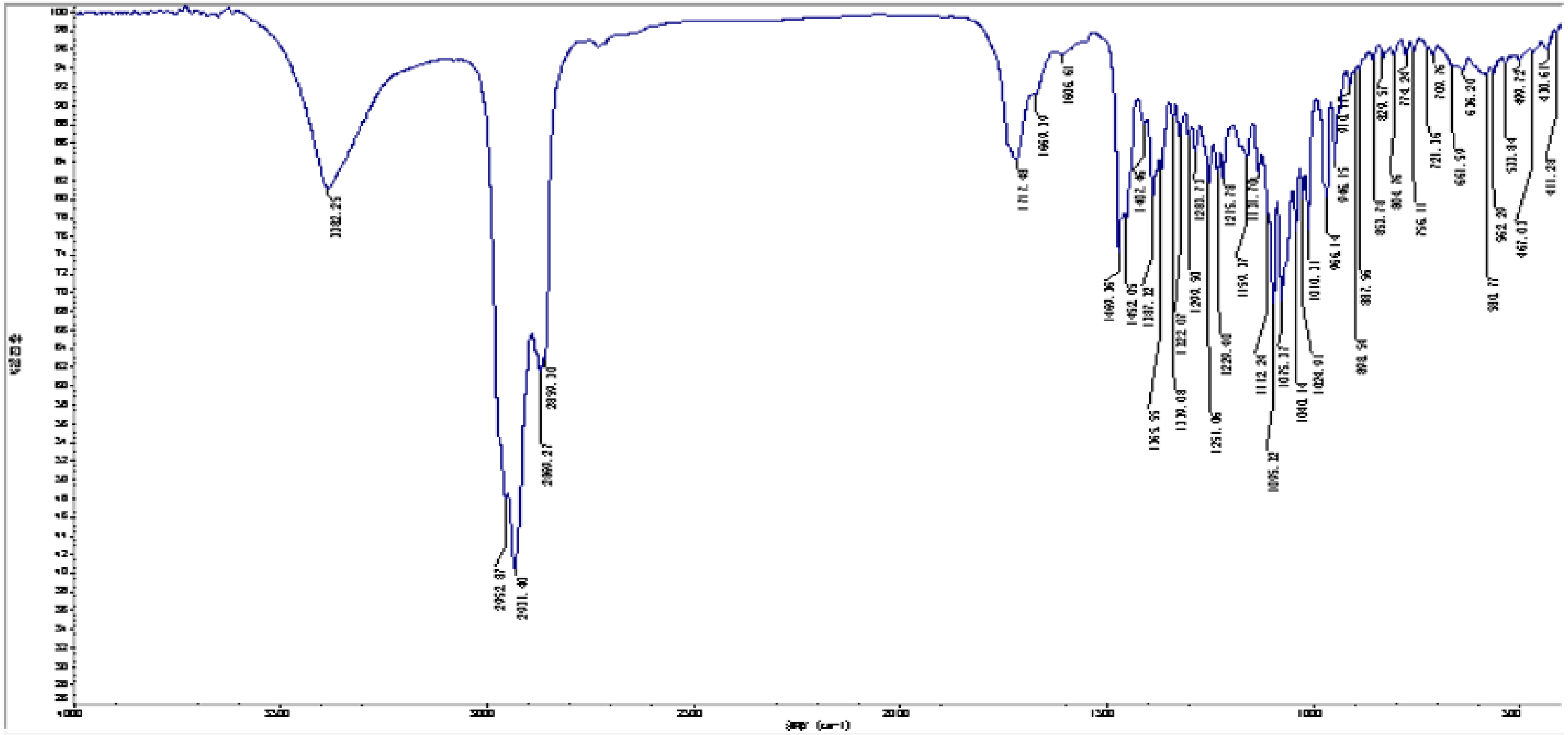

Sample Name: YBJ-58

KBr压片

(Measured on)

采集时间: 星期四 10月 14 15:31:30 2021 (GMT+08:00)

仪器型号: NICOLET iS10 (Instrument)

(Sample scan) 样品扫描次数: 16

背景扫描次数: 16

(Resolution) 分辨率: 4.000

采样增益: 1.0

动镜速度: 0.4747

(Acquisition) 光阑: 80.00

The UV spectrum of compound **1**

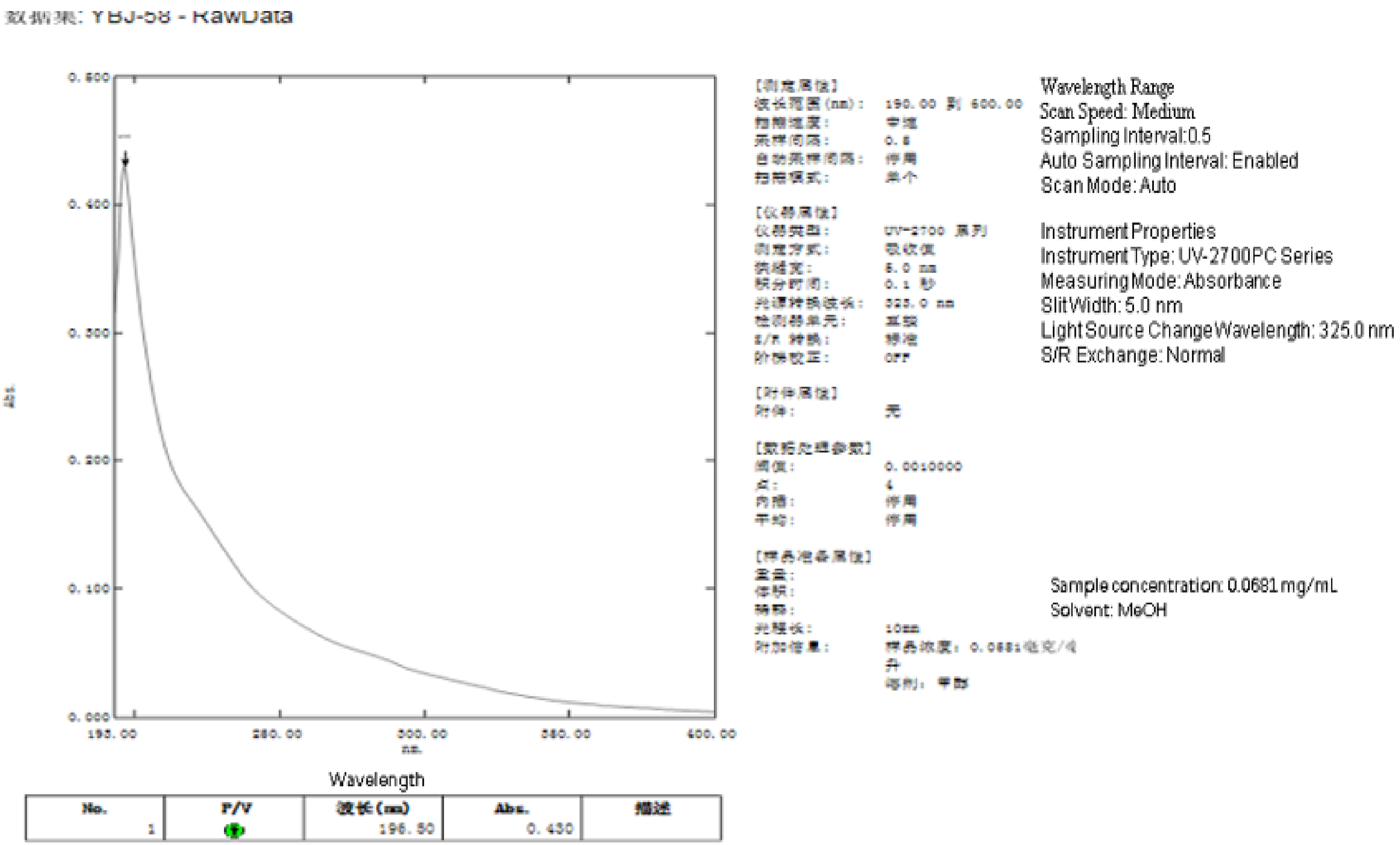

The ^{1}H NMR spectrum of compound **3**

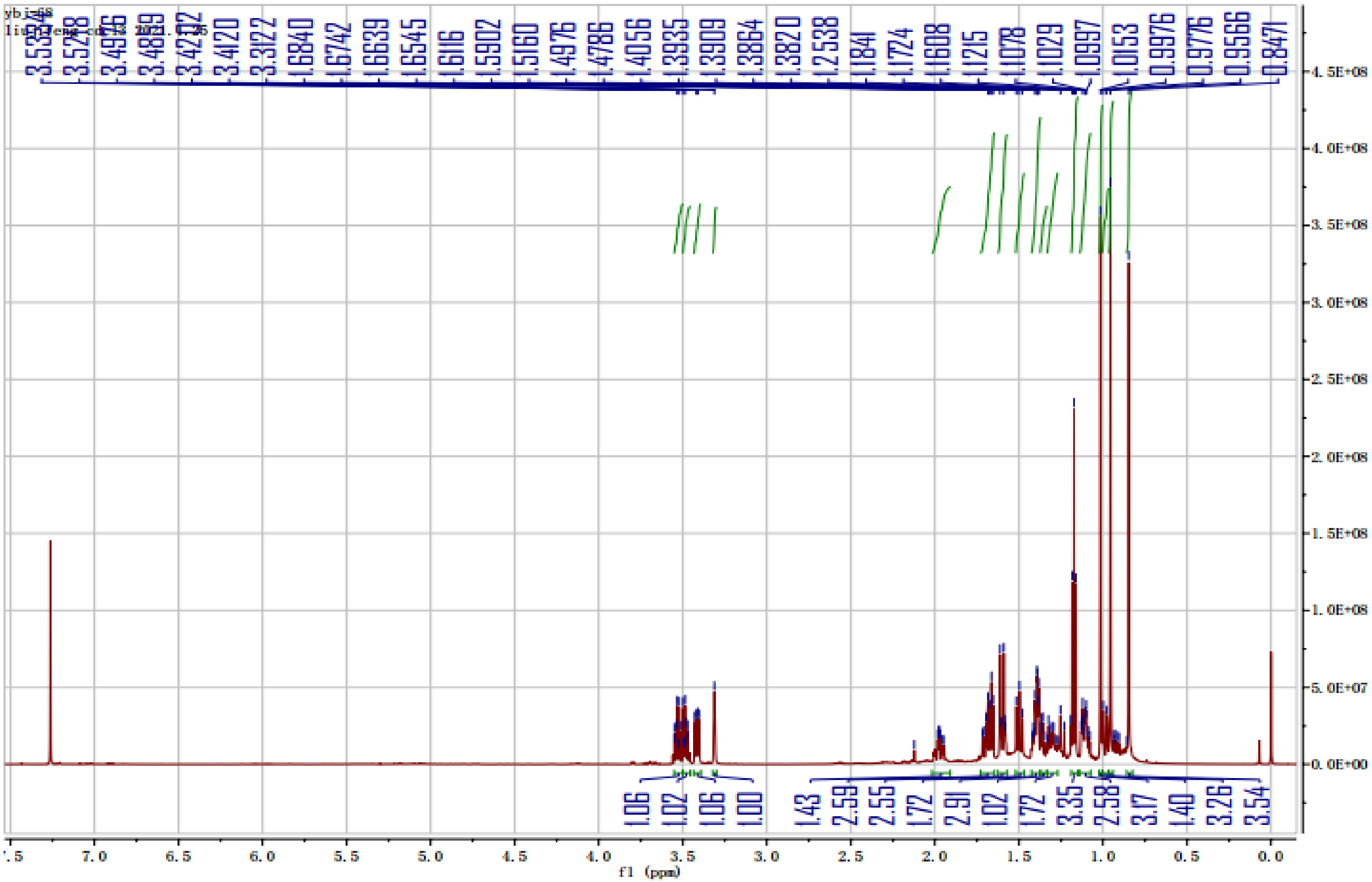

The ^{1}H NMR spectrum of compound **3**

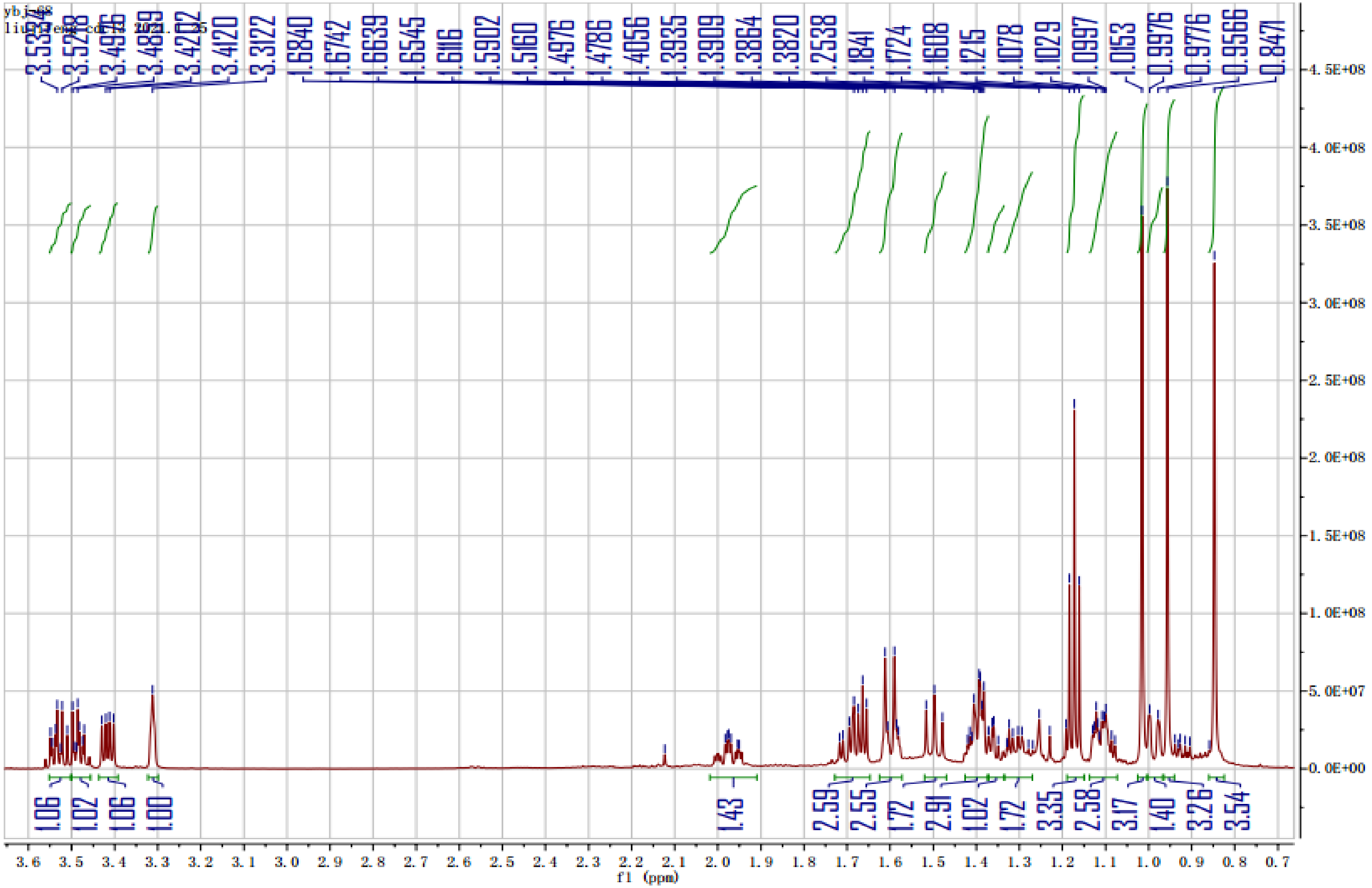

The ^{13}C NMR spectrum of compound **3**

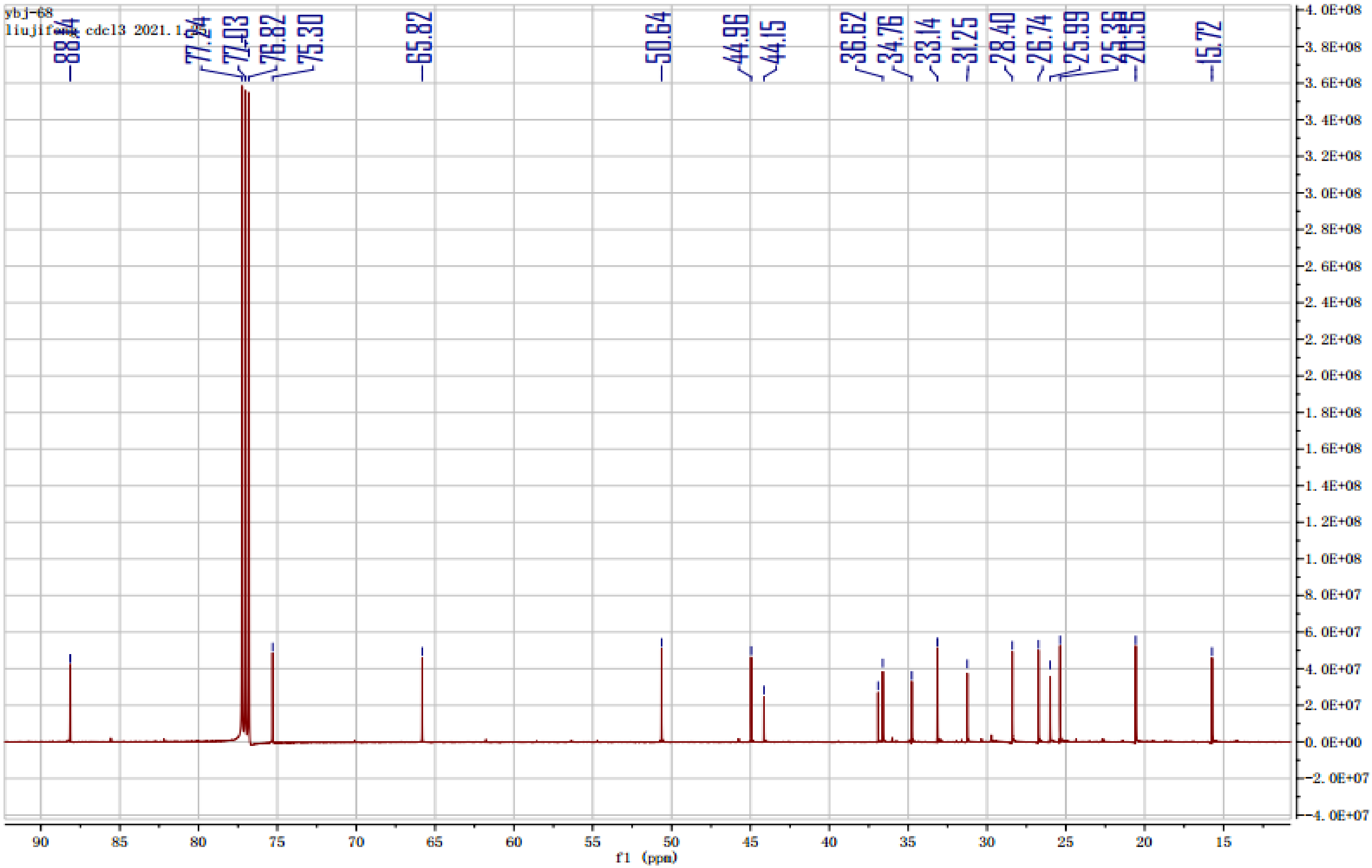

The DEPT-135 spectrum of compound **3**

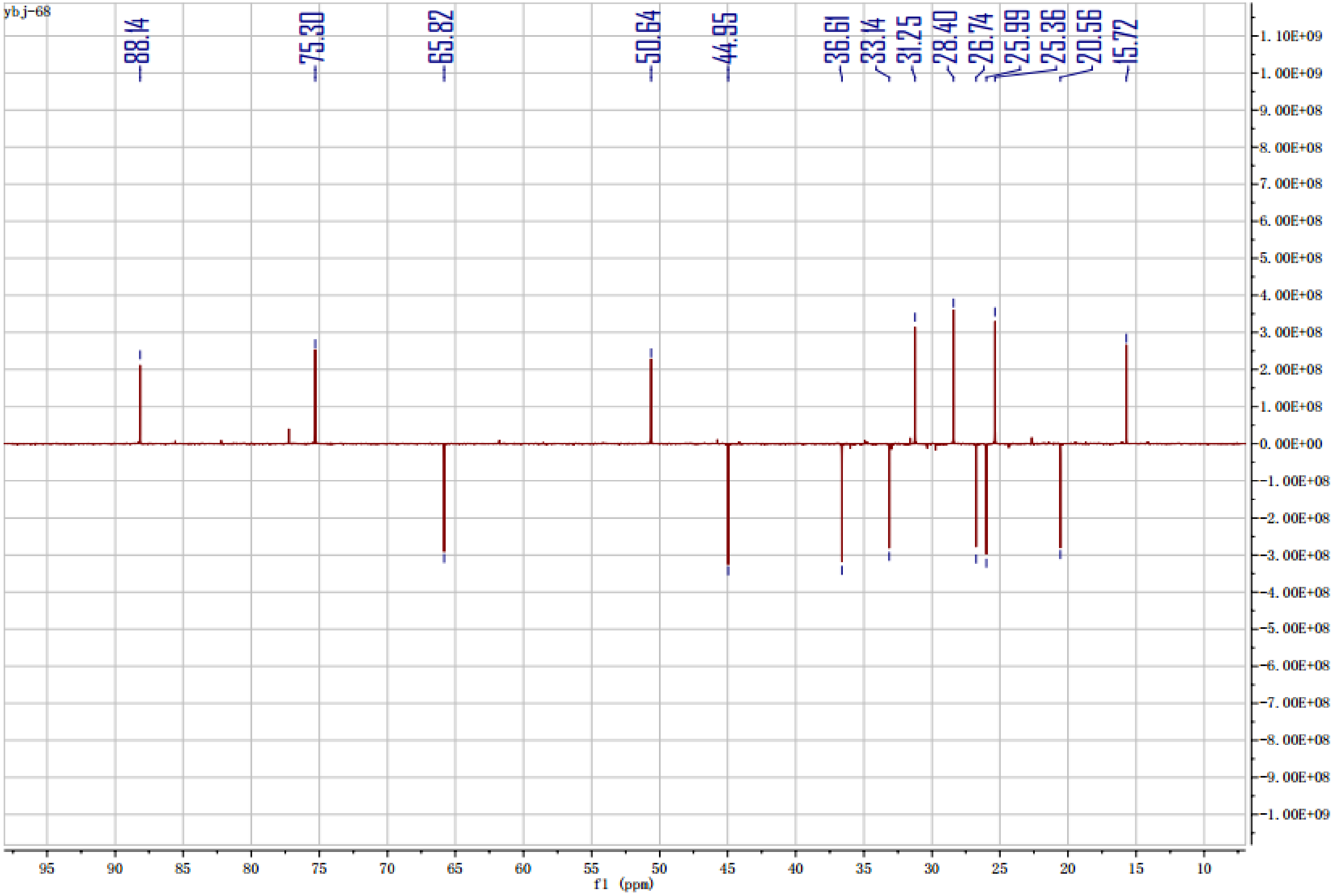

The DEPT-90 spectrum of compound **3**

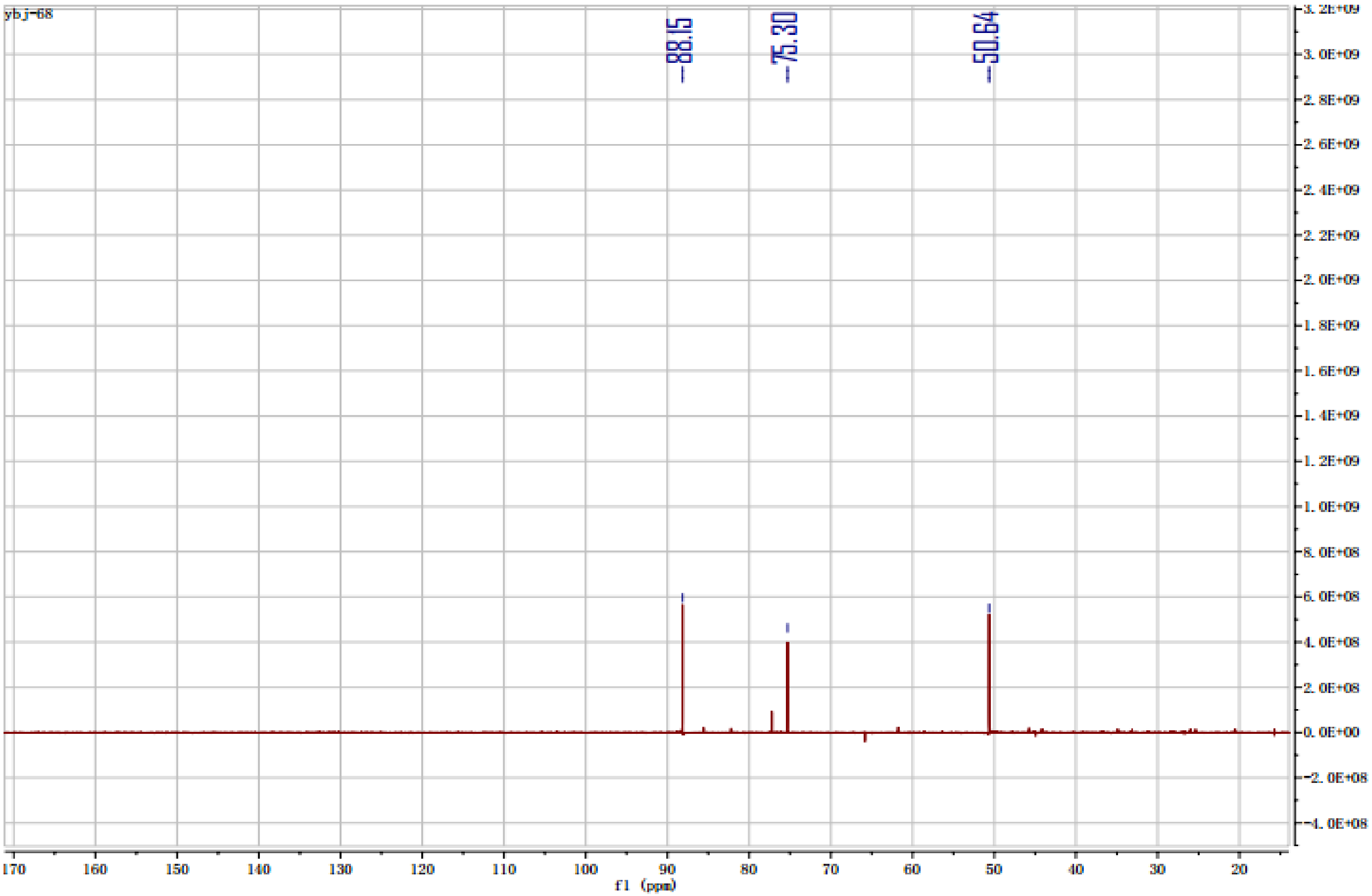

The HSQC spectrum of compound **3**

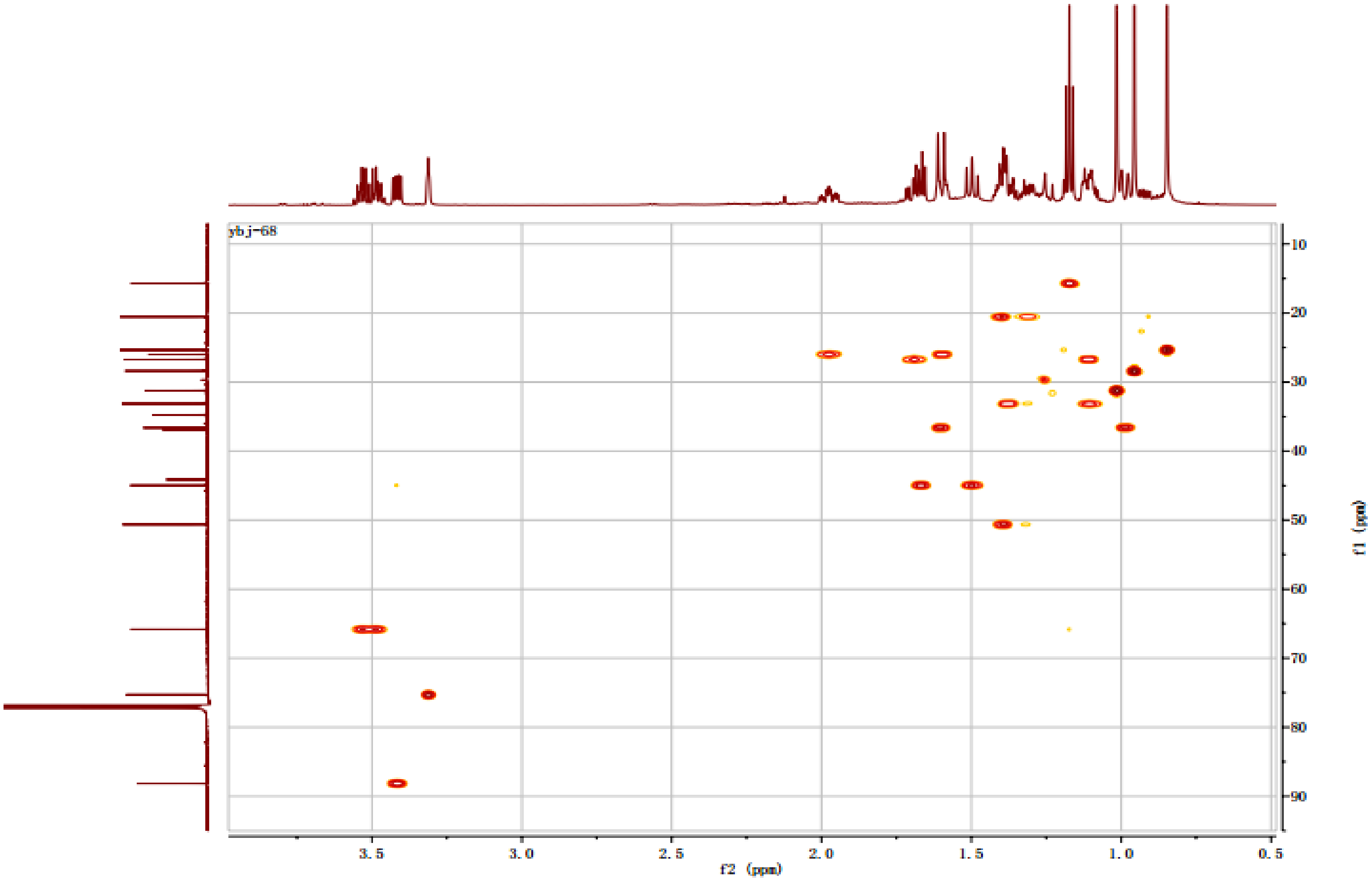

The HMBC spectrum of compound **3**

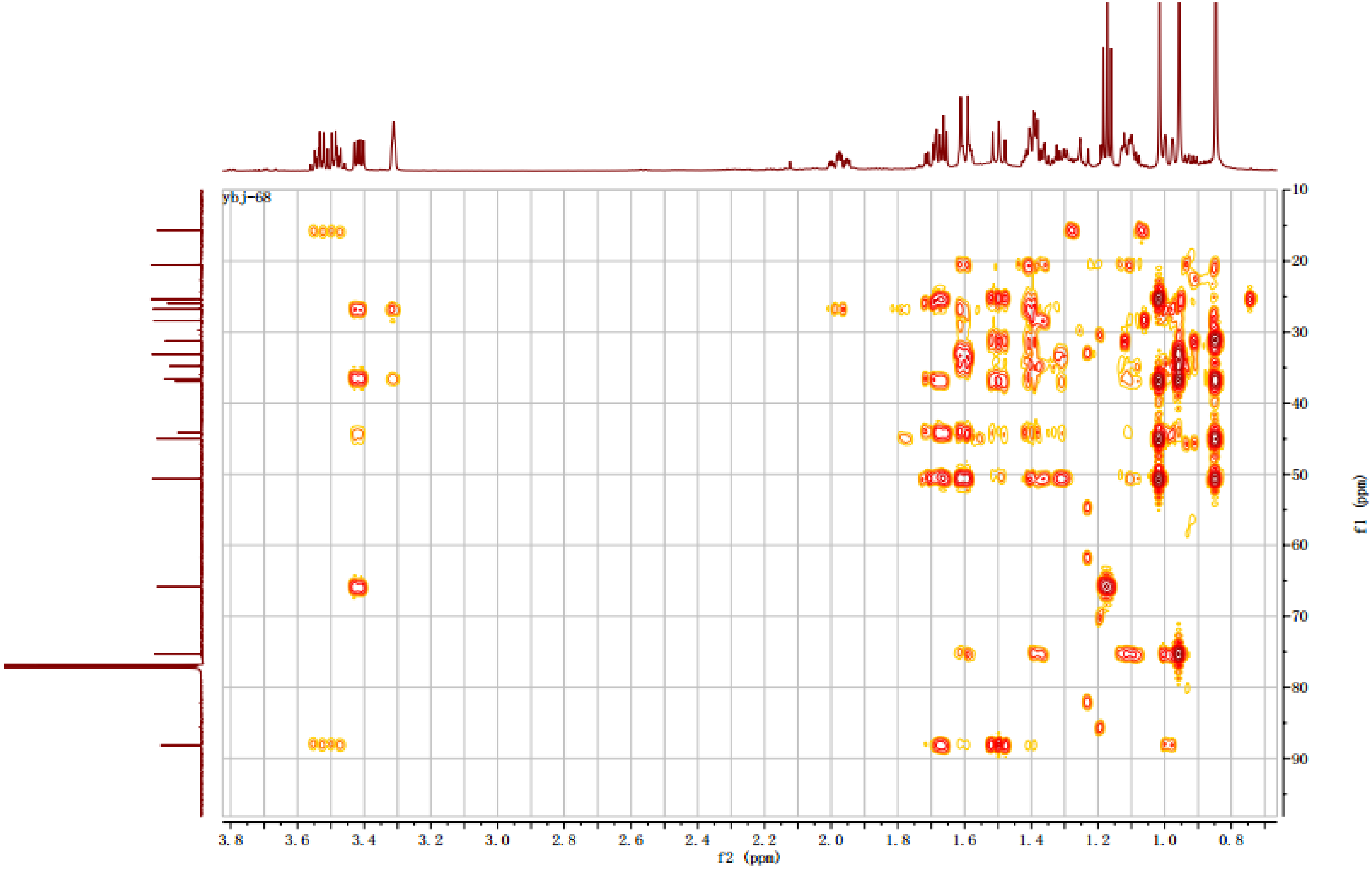

The COSY spectrum of compound **3**

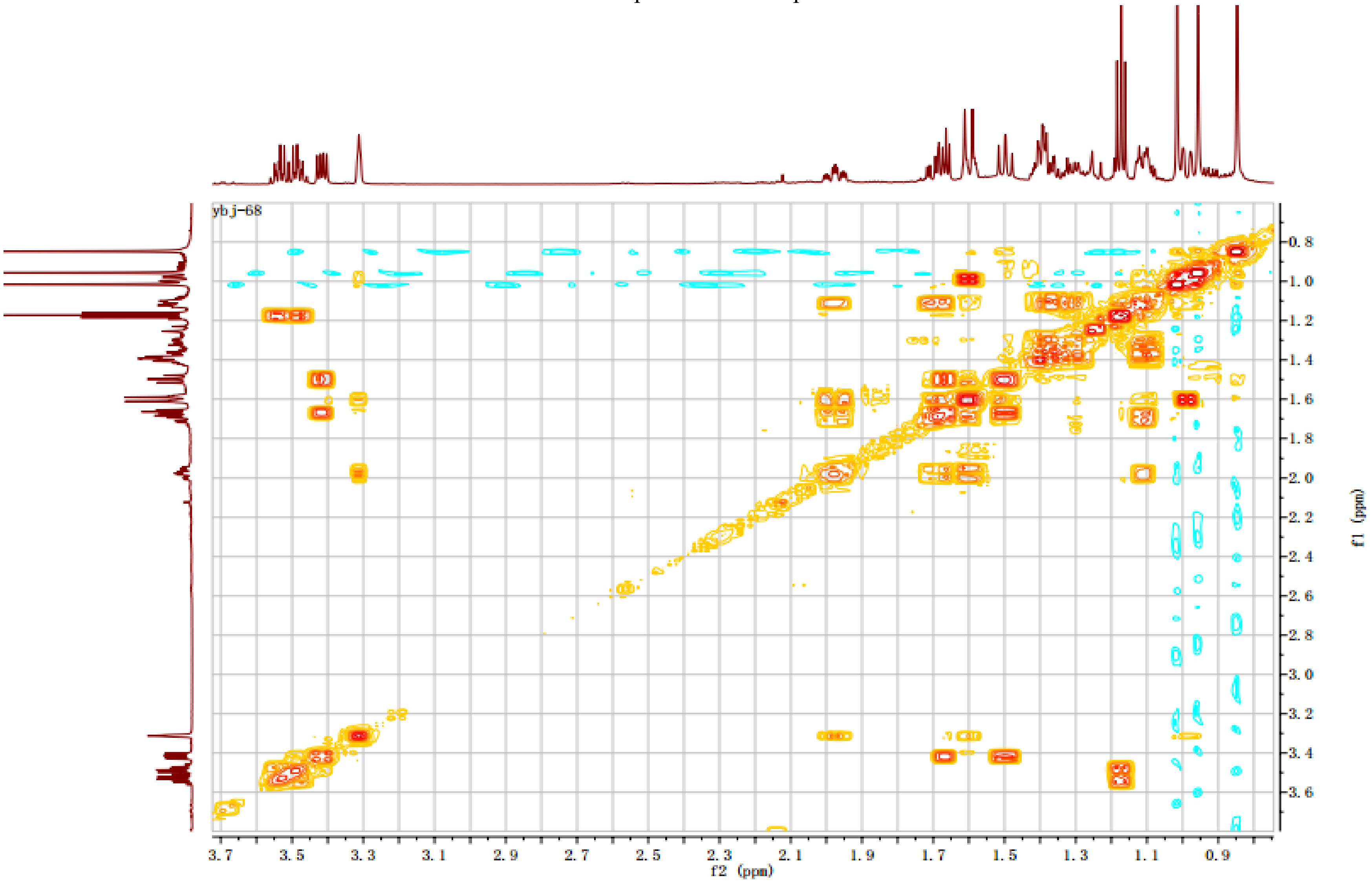

The ROESY spectrum of compound **3**

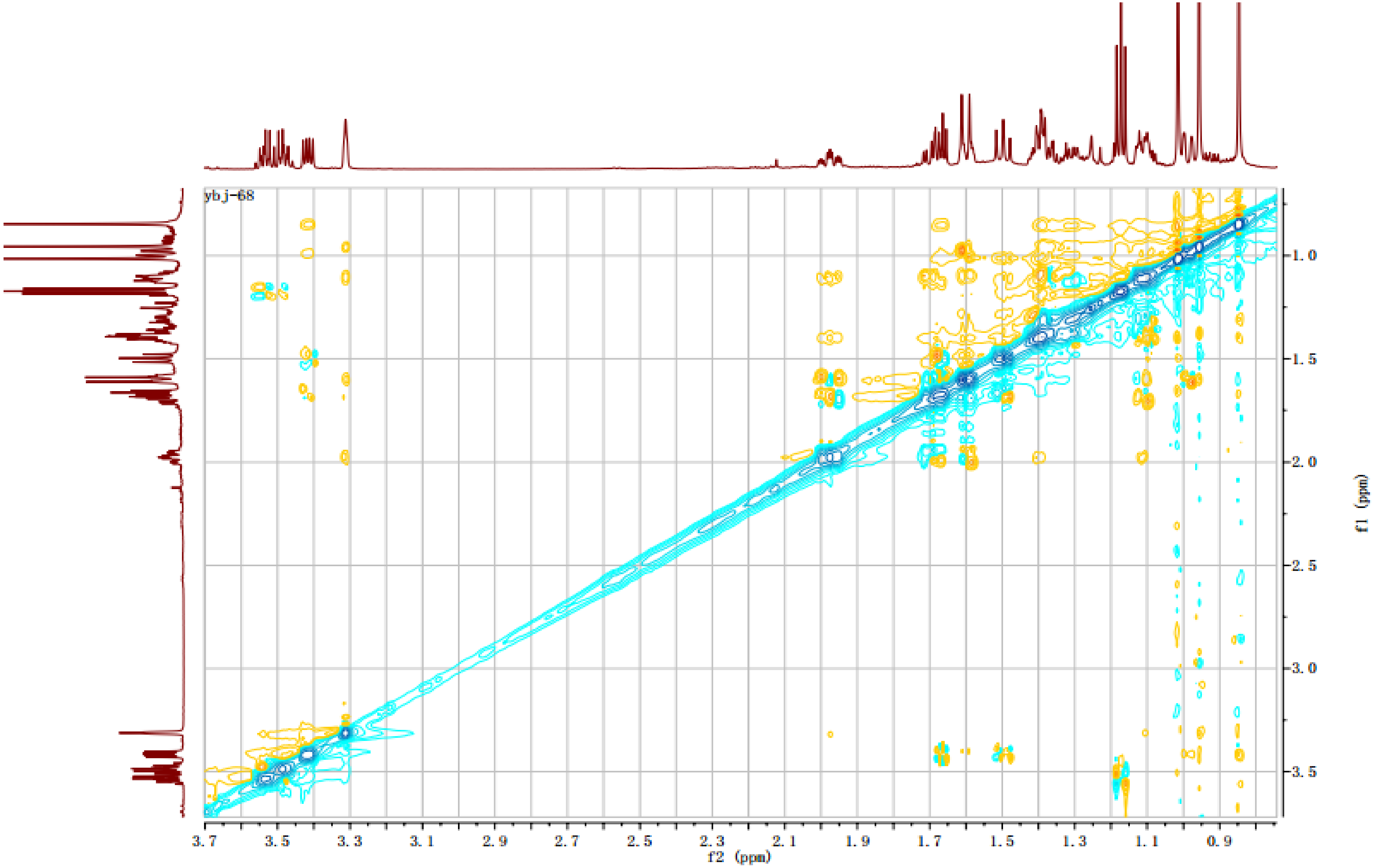

The HR-ESI-MS spectrum of compound **3**

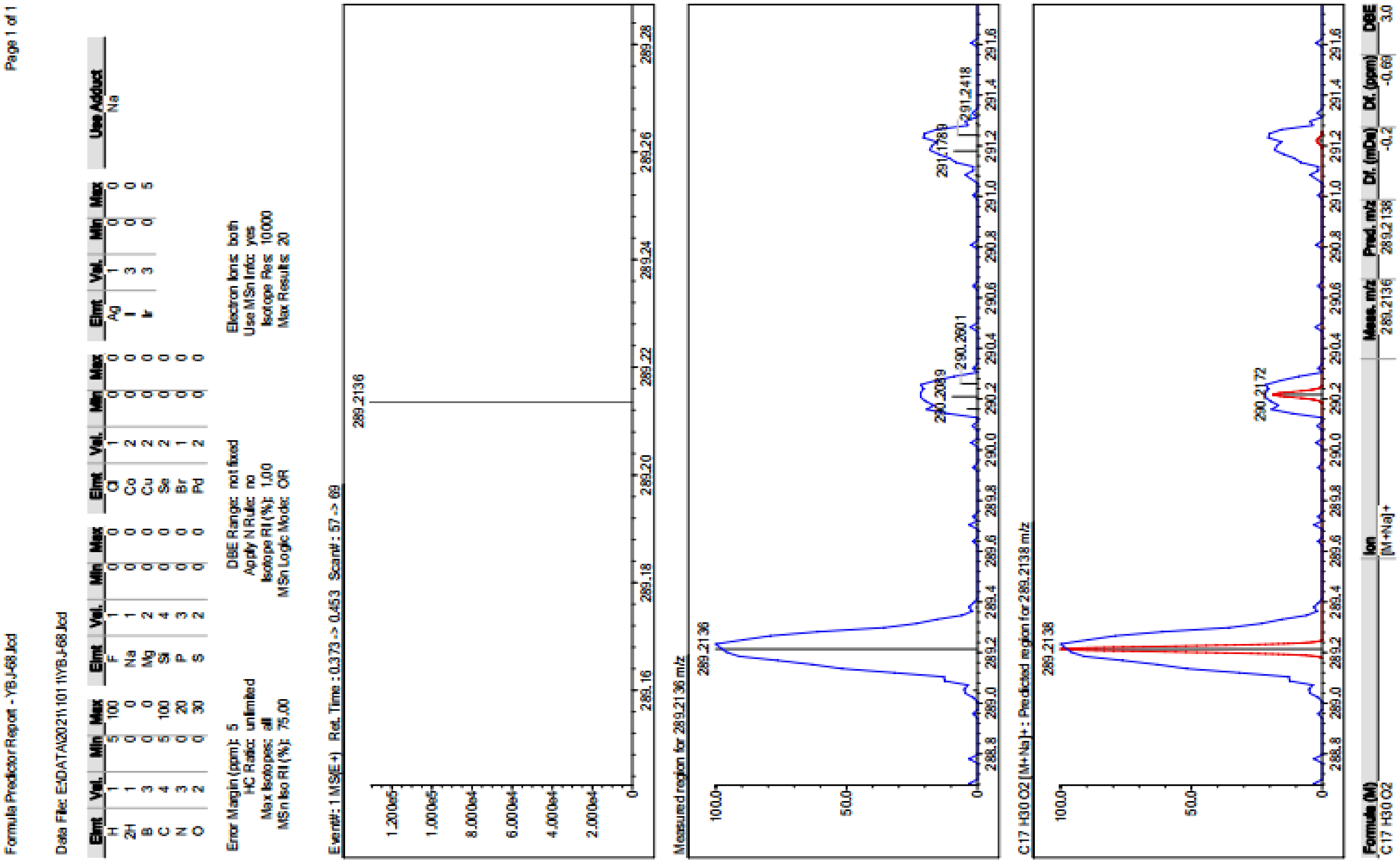

The IR (KBr) spectrum of compound **3**

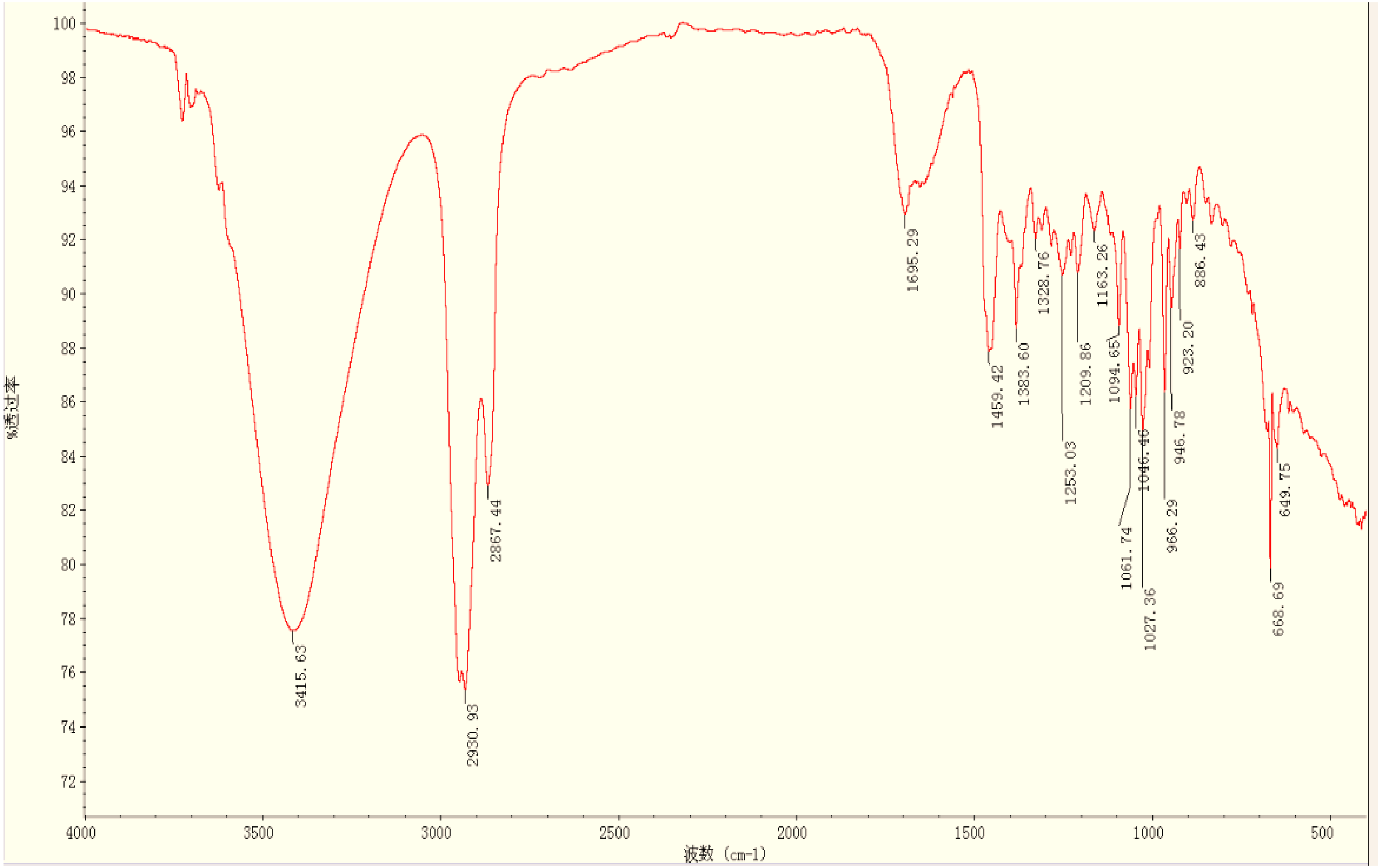

The UV spectrum of compound **3**

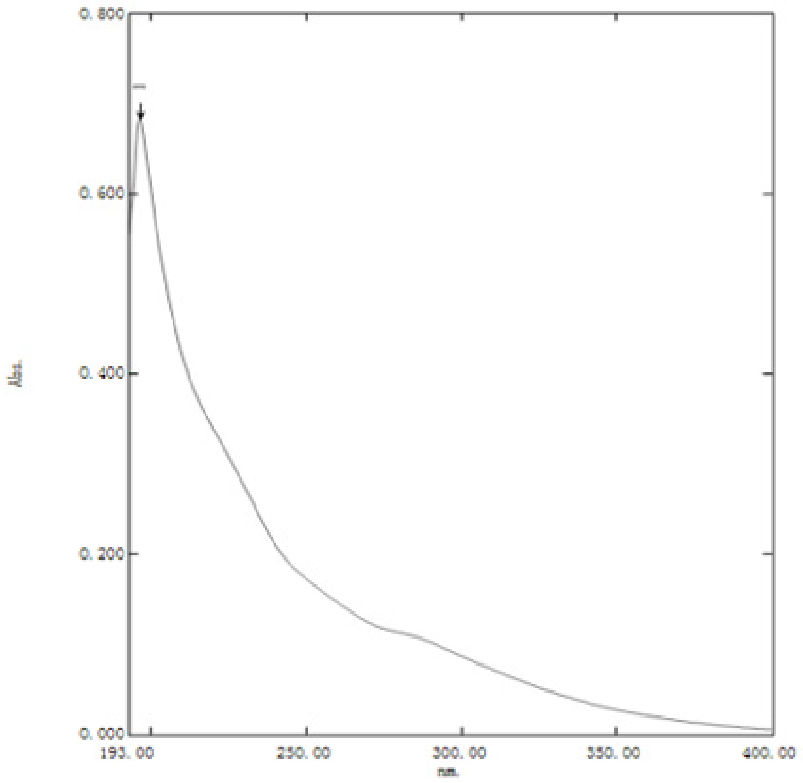

[测定属性]
波长范围(nm): 190.00 到 600.00
扫描速度: 中速
采样间隔: 0.5
自动采样间隔: 停用
扫描模式: 单个

[仪器属性]
仪器类型: UV-2700 系列
测定方式: 吸收值
狭缝宽: 5.0 nm
积分时间: 0.1 秒
光源转换波长: 323.0 nm
检测器单元: 直接
S/R 转换: 标准
阶梯校正: OFF

[附件属性]
附件: 无

[数据处理参数]
阈值: 0.0010000
点: 4
内插: 停用
平均: 停用

[样品准备属性]
重量:
体积:
稀释:
光程长: 10mm
附加信息: 样品浓度: 0.1072毫克/毫升
溶剂: 甲醇

Wavelength Range:1
Scan Speed: Medium
Sampling Interval:0.5
Auto Sampling Interval: Enabled
Scan Mode: Auto

Instrument Properties
Instrument Type: UV-2700PC Series
Measuring Mode: Absorbance
Slit Width: 5.0 nm
Light Source Change Wavelength: 325.0 nm
S/R Exchange: Normal

Sample concentration: 0.1072 mg/mL
Solvent: MeOH

Wavelength

No.	P/V	波长(nm)	Abs.	描述
1	+	196.50	0.683	

The ^{1}H NMR spectrum of compound **5**

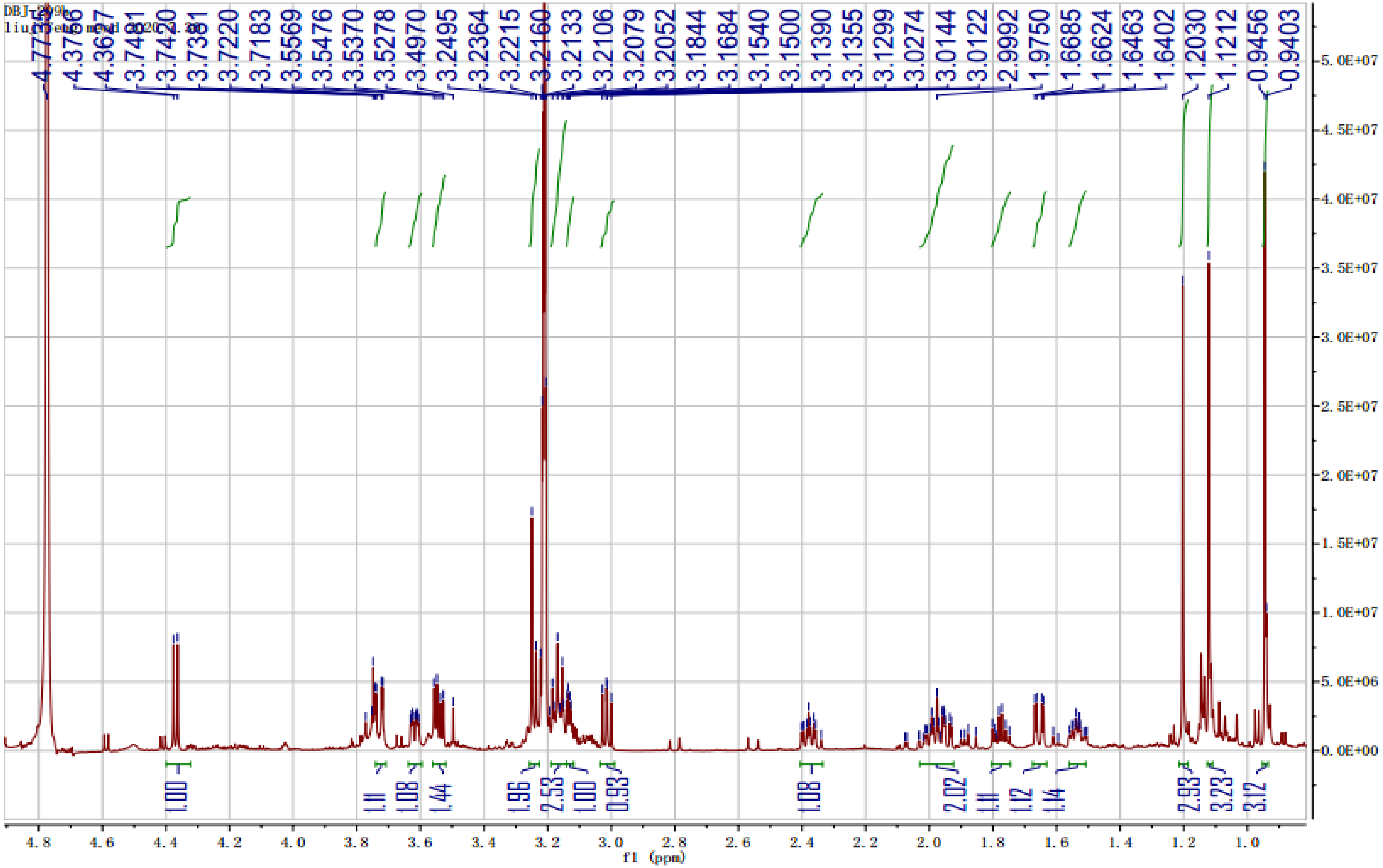

The ^{13}C NMR spectrum of compound **5**

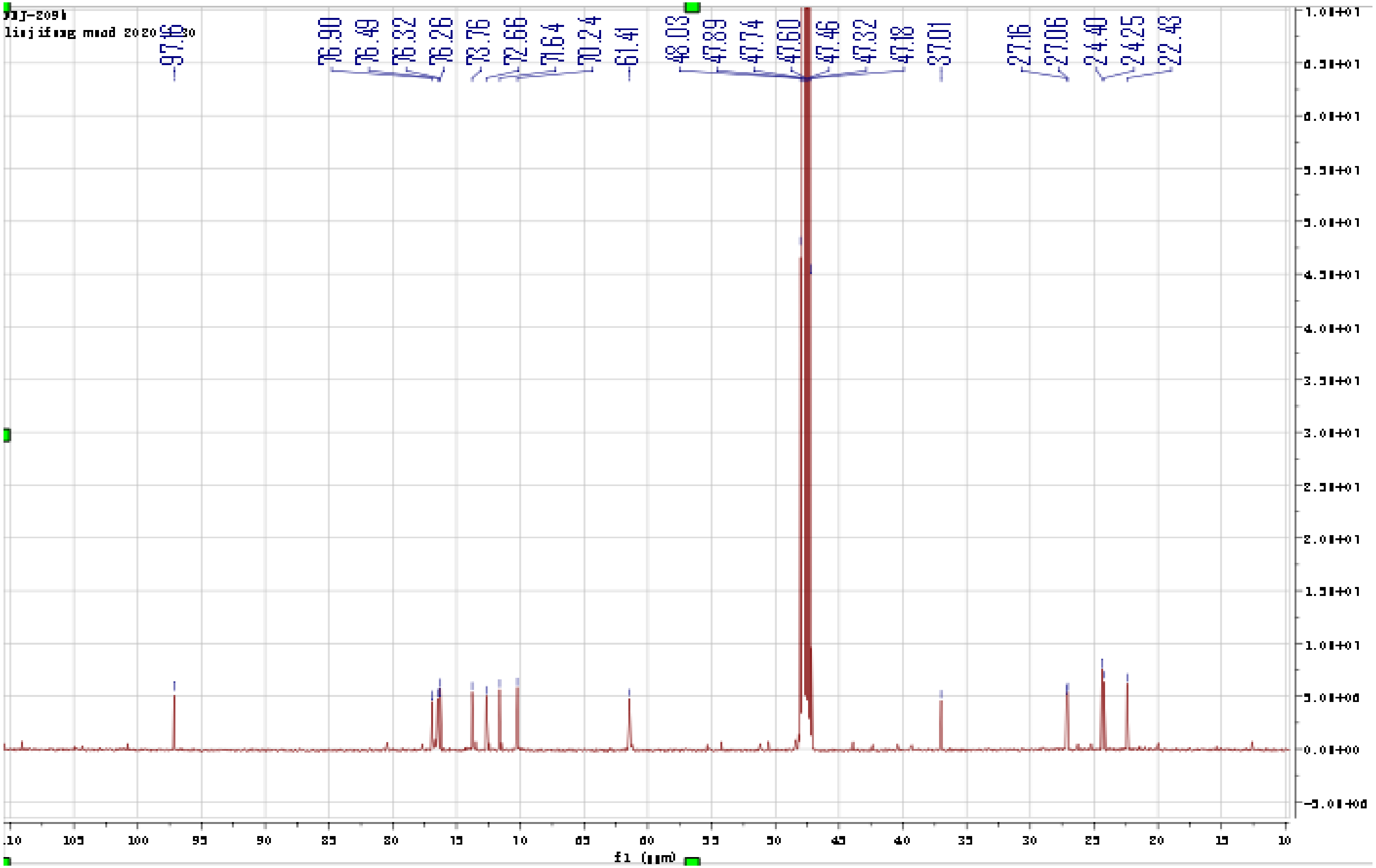

The DEPT-135 spectrum of compound **5**

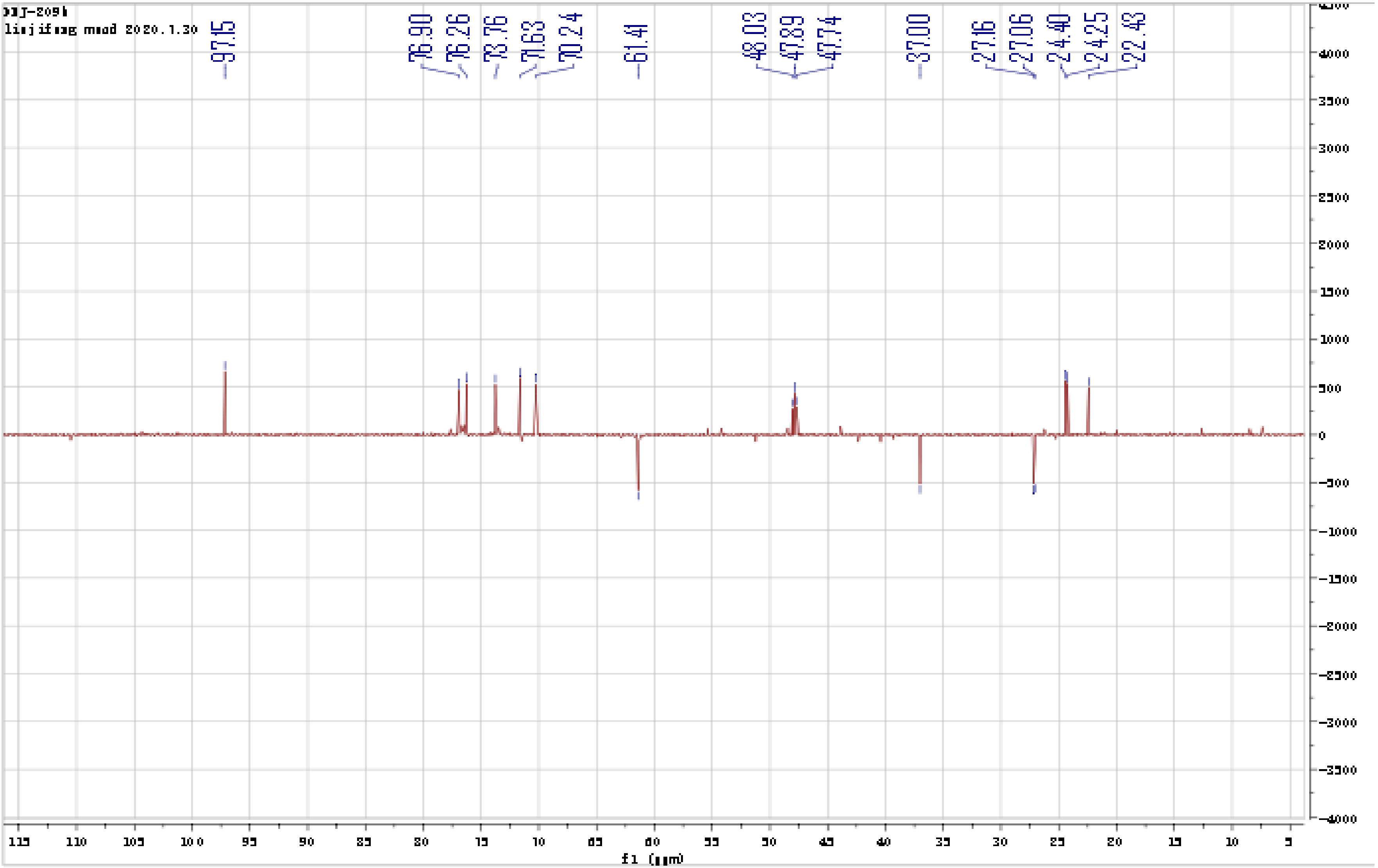

The DEPT-90 spectrum of compound **5**

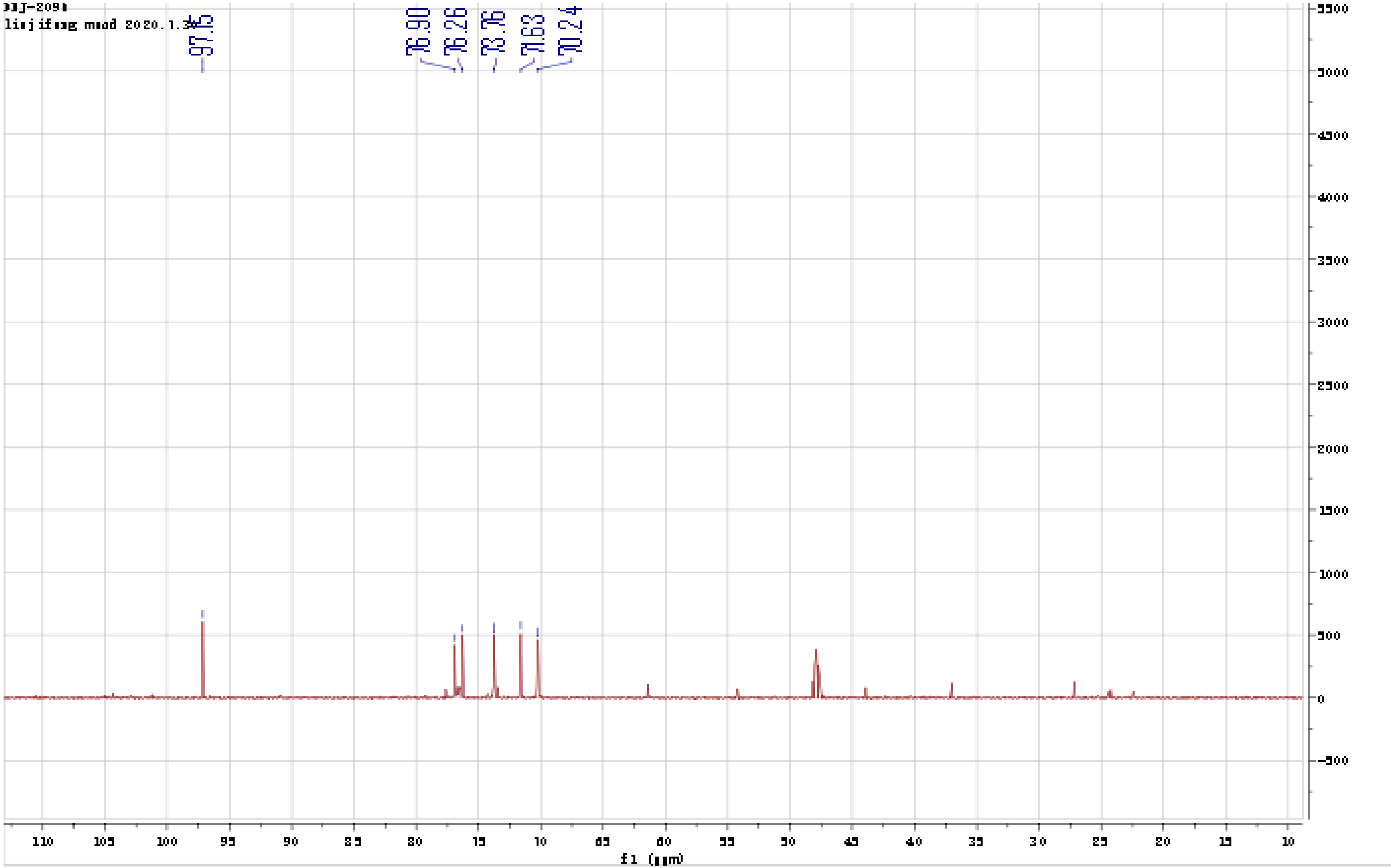

The HSQC spectrum of compound **5**

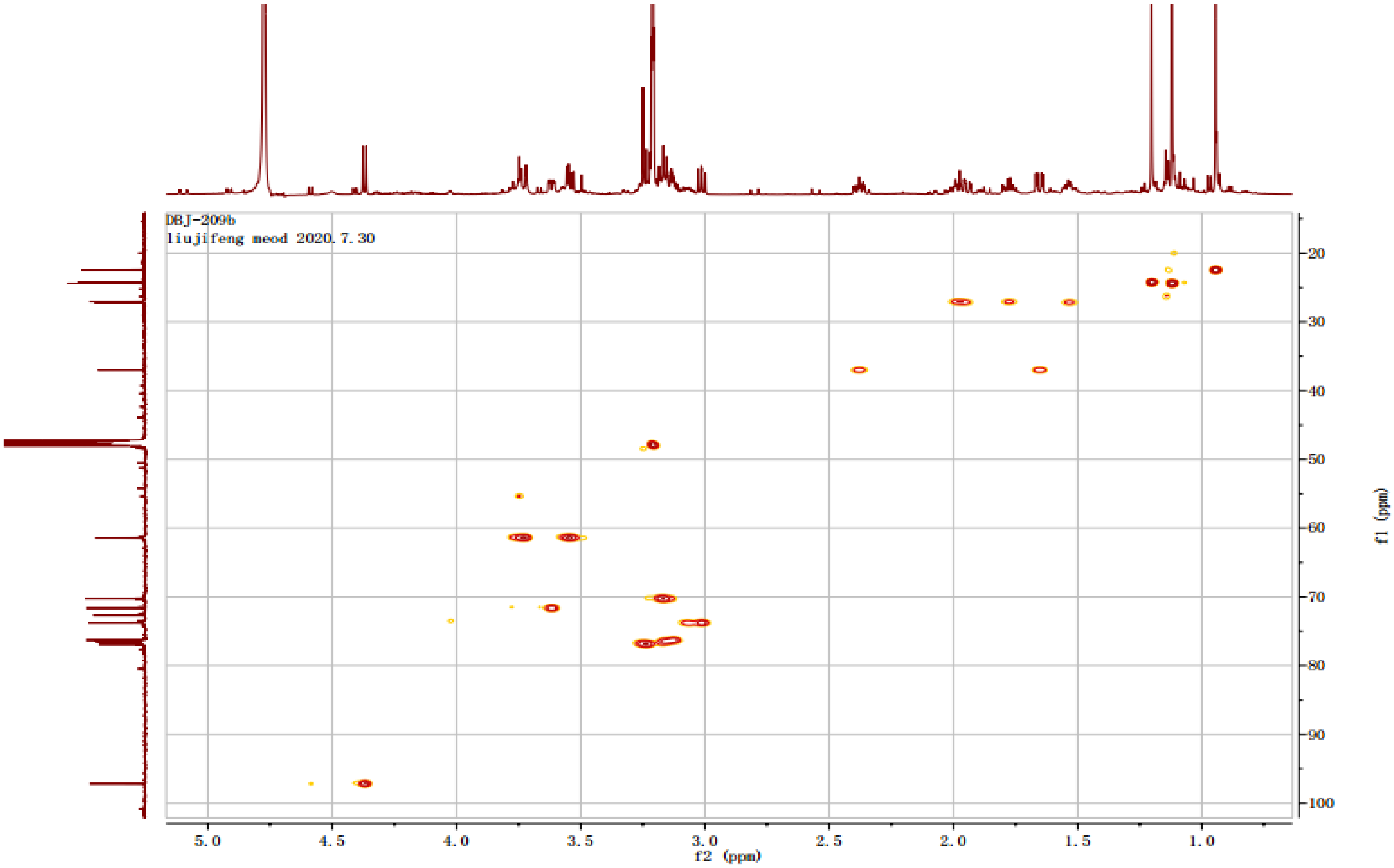

The HMBC spectrum of compound **5**

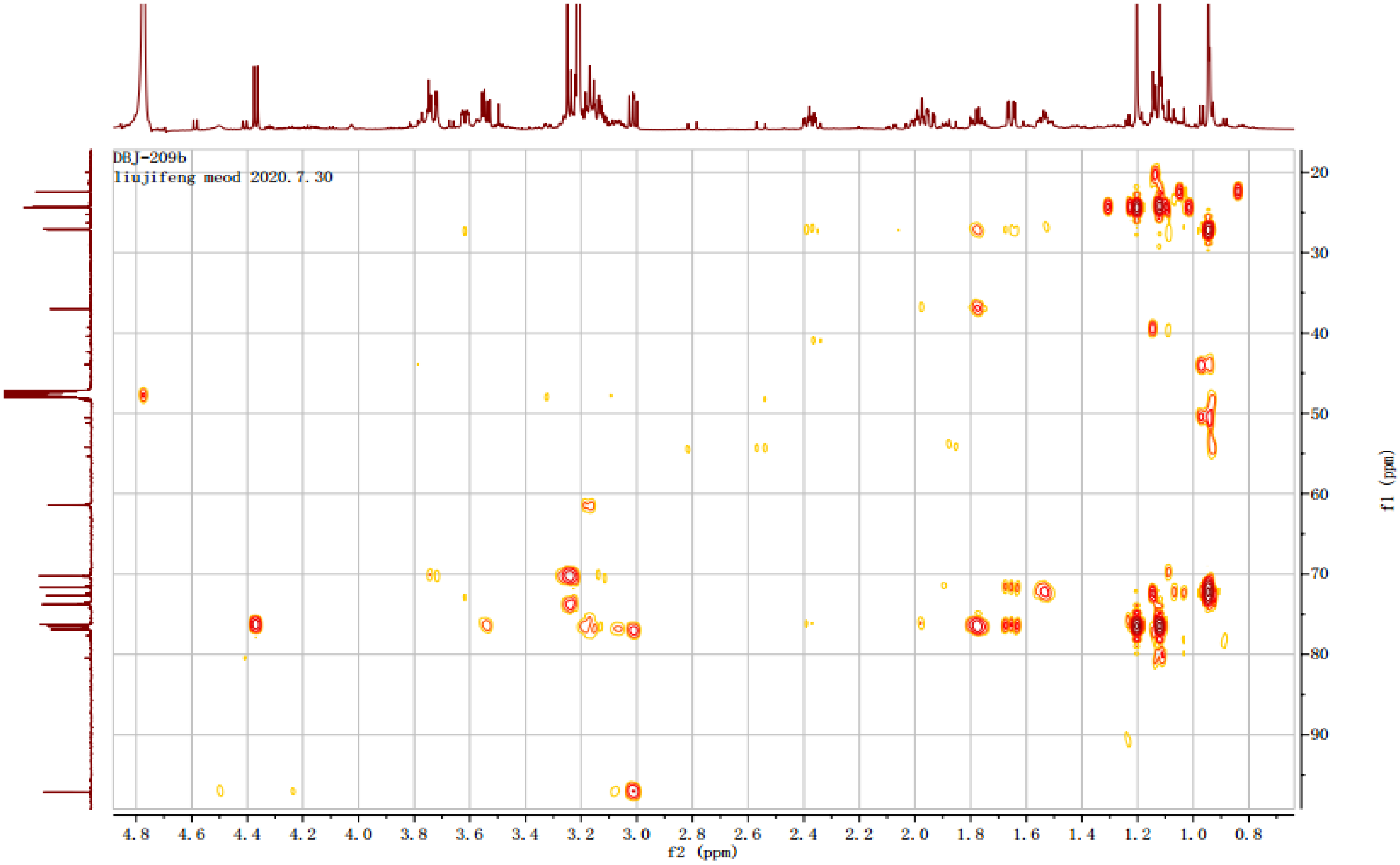

The ^1H-^1H COSY spectrum of compound **5**

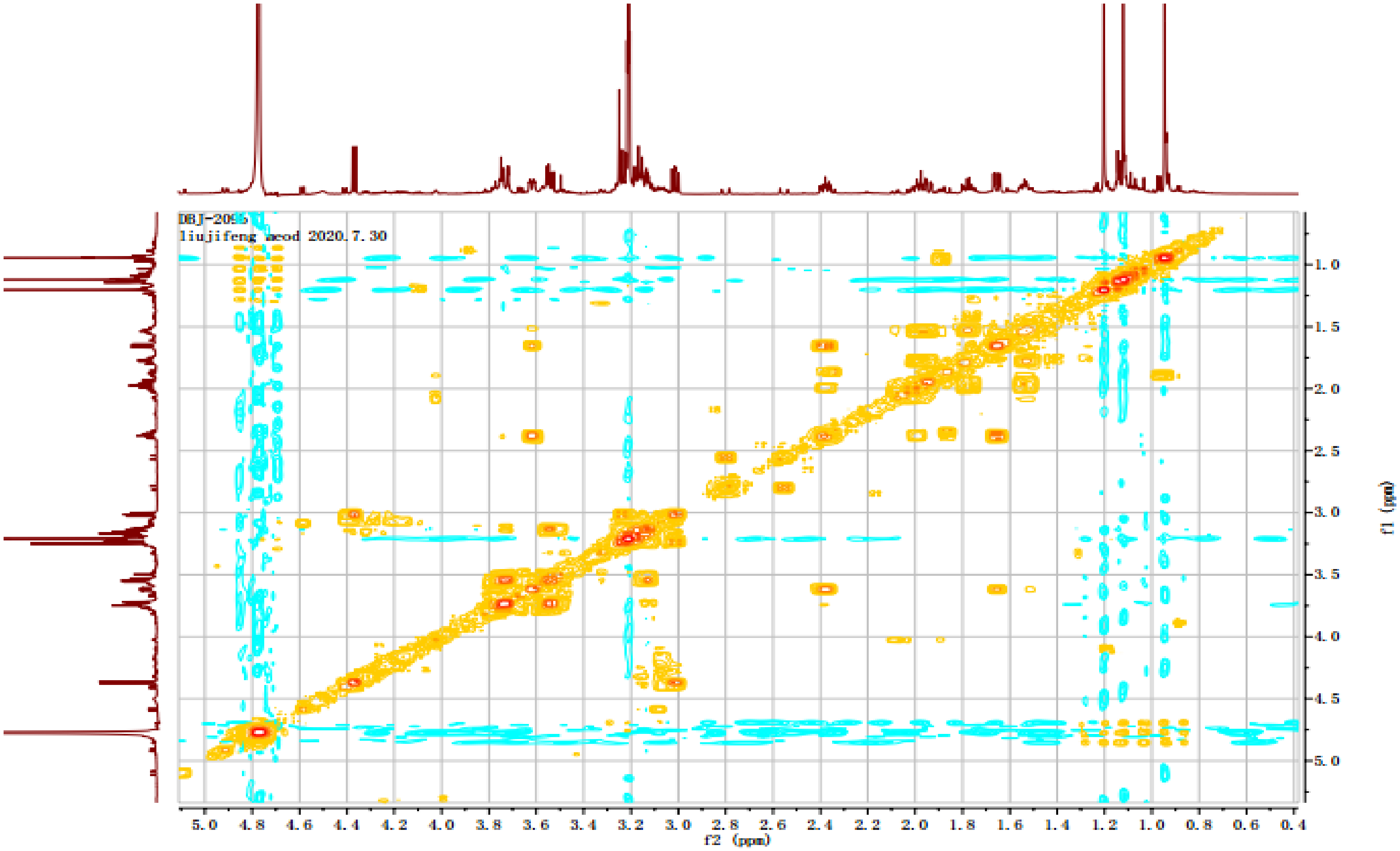

The ROESY spectrum of compound **5**

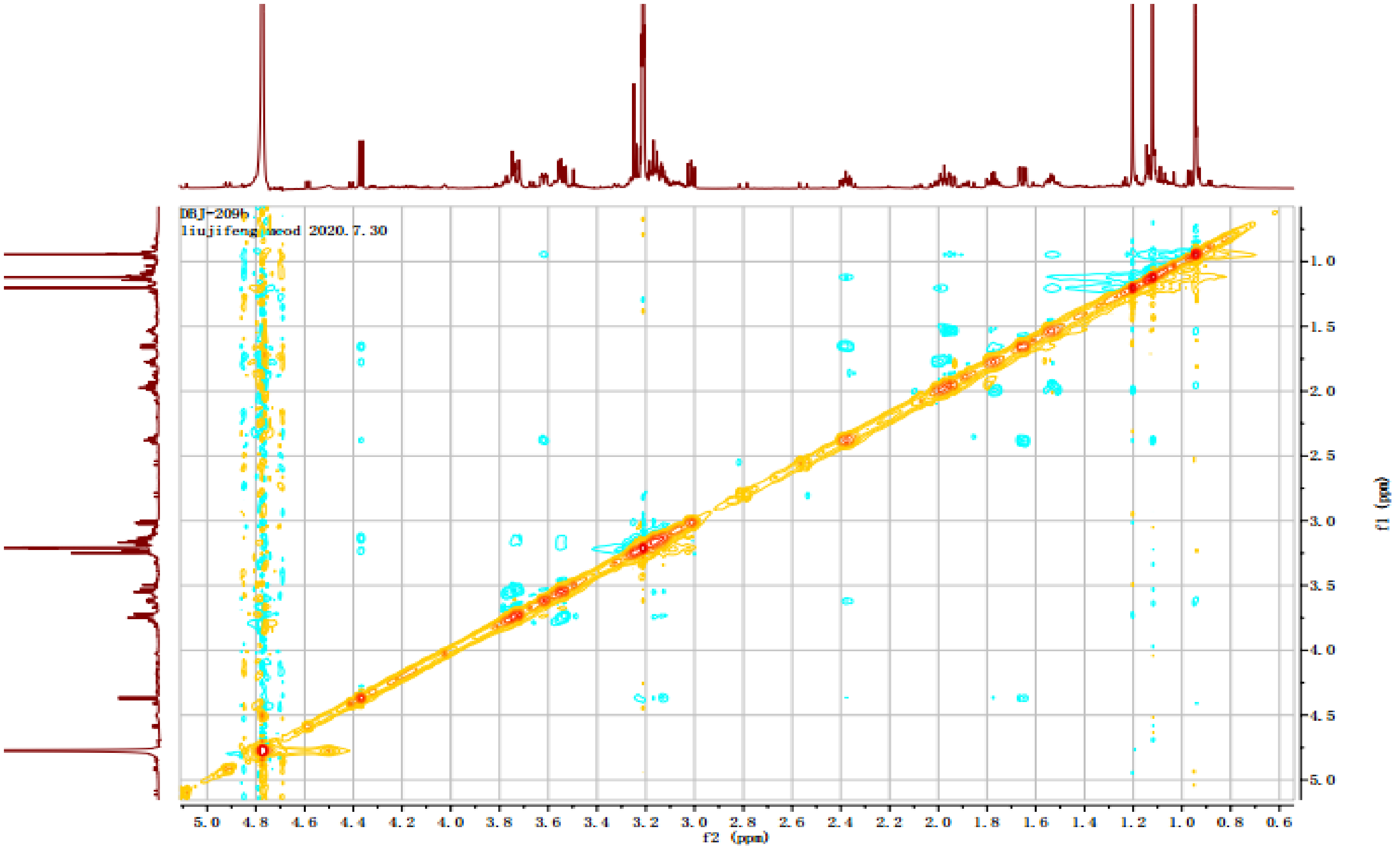

The HRESI spectrum of compound **5**

Formula Predictor Report - DBJ-209B_19.lcd

Data File: D:\wendang\2020-10-06\DBJ-209B_19.lcd

Elmt	Val.	Min	Max	Elmt	Val.	Min	Max	Use Adduct
H	1	0	300	O	2	0	50	H
C	4	0	100	S	2	0	0	Na
N	3	0	0	Br	1	0	0	

Error Margin (mDa): 20.0
HC Ratio: unlimited
Max Isotopes: all
MSn Iso RI (%): 75.00

DBE Range: 0.0 - 1000.0
Apply N Rule: yes
Isotope RI (%): 1.00
MSn Logic Mode: AND

Electron Ions: both
Use MSn Info: no
Isotope Res: 10000
Max Results: 500

Event#: 1 MS(E+) Ret. Time : 0.430 Scan# : 87

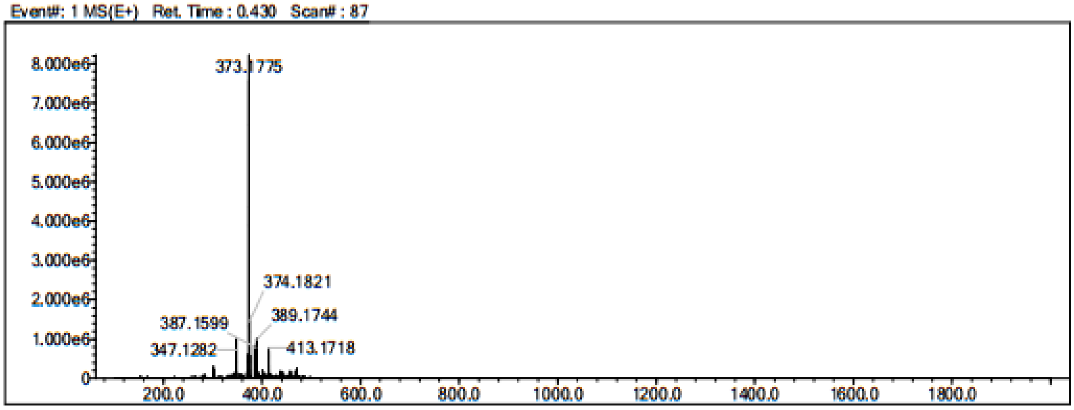

Measured region for 389.1744 m/z

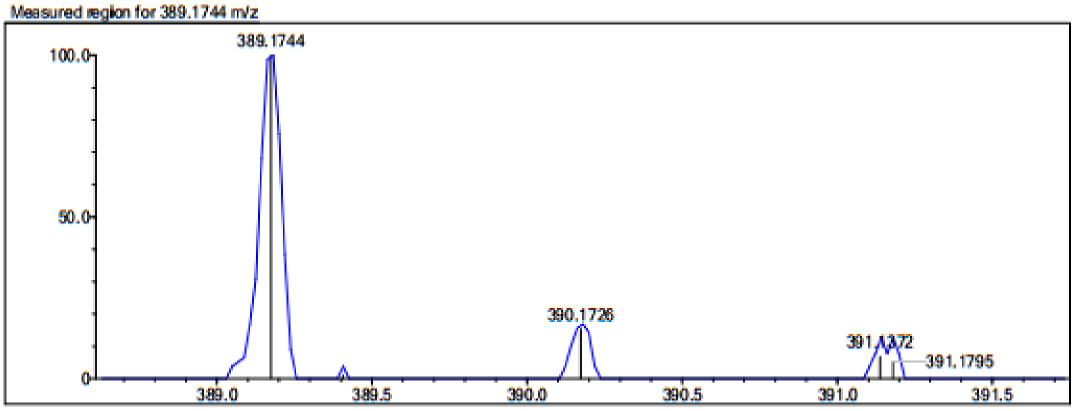

C16 H30 O9 [M+Na]+ : Predicted region for 389.1782 m/z

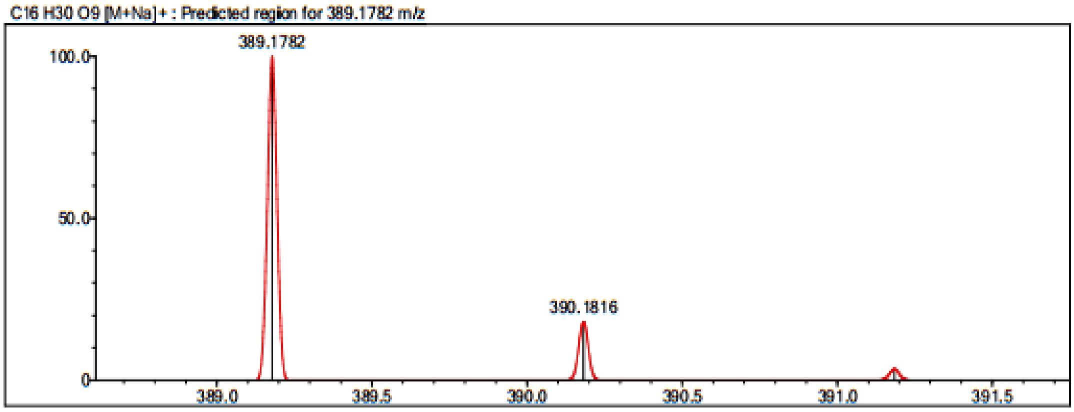

Rank	Score	Formula (M)	Ion	Meas. m/z	Pred. m/z	Df. (mDa)	Df. (ppm)	Iso	DBE
3	21.69	C16 H30 O9	[M+Na]+	389.1744	389.1782	-3.8	-9.76	51.15	2.0

The IR (KBr) spectrum of compound **5**

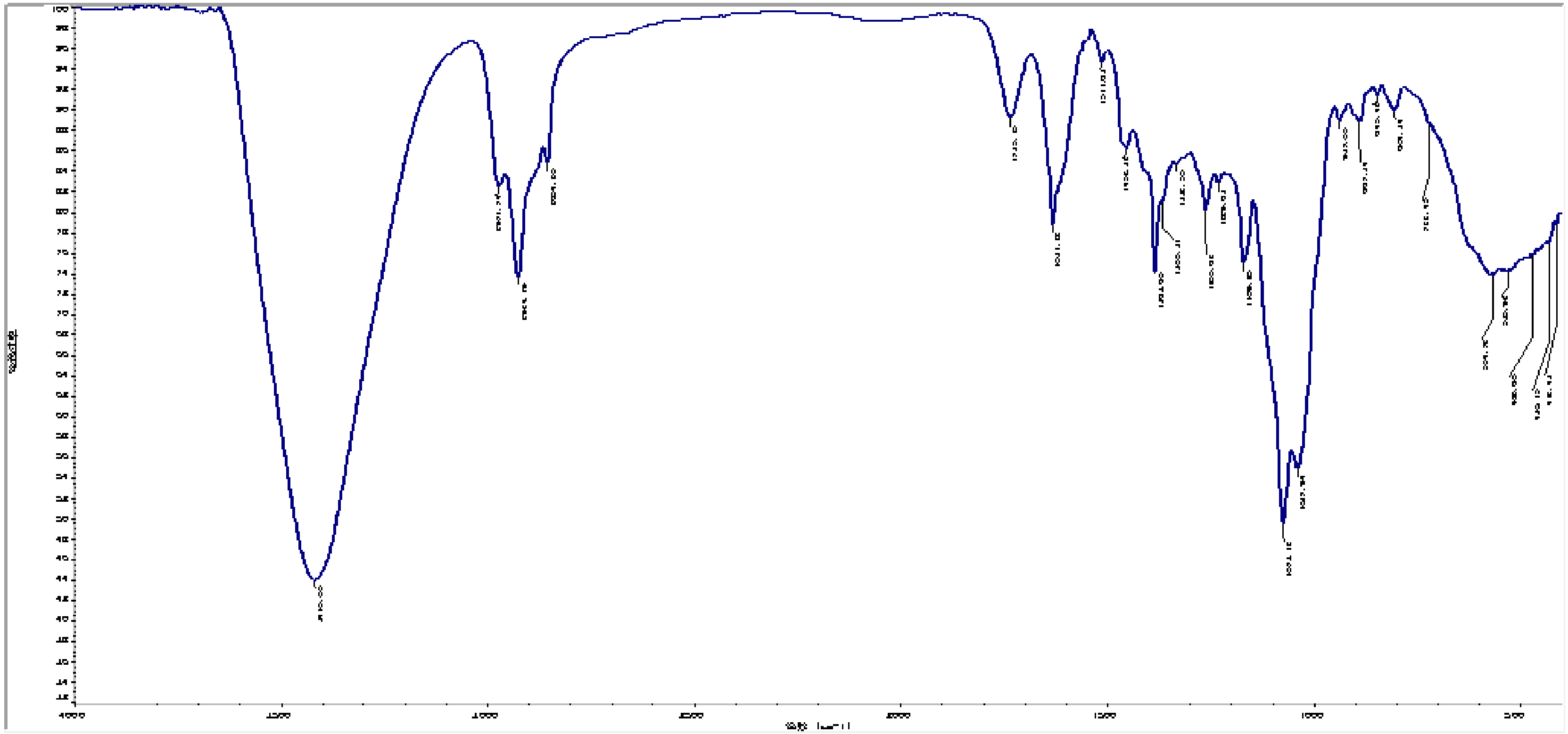

Sample Name: DBJ-209B
KBr压片
采集时间: 星期四 10月 22 16:24:04 2020 (GMT+08:00)(Measured on)
仪器型号：NICOLET iS10 (Instrument)
Software version: OMNIC 9.8.372

(Sample scan) 样品扫描次数: 16
背景扫描次数: 16
(Resolution) 分辨率: 4.000
采样增益: 1.0
动镜速度: 0.4747
(Acquisition) 光阑: 80.00

The UV spectrum of compound **5**

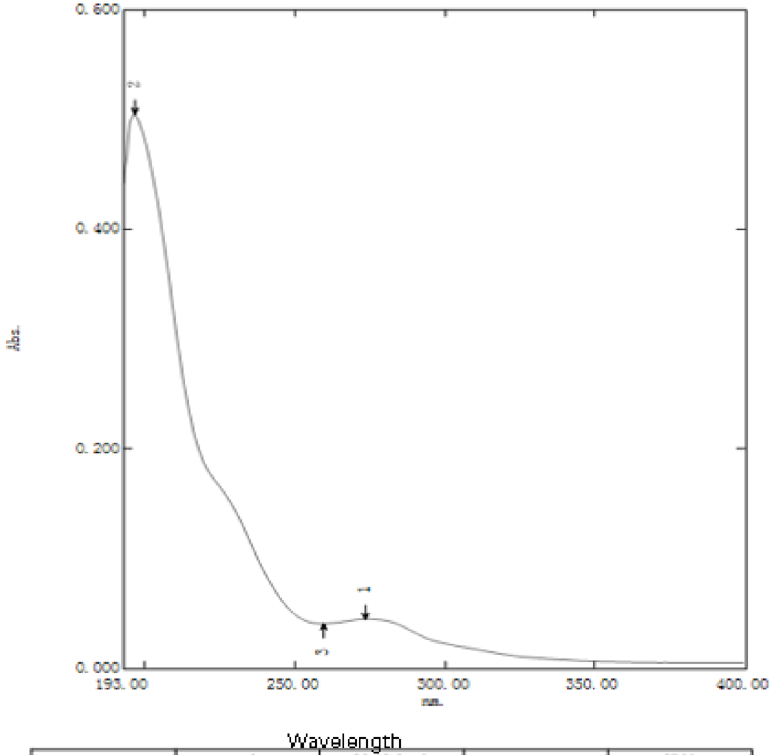

[测定属性]
波长范围(nm)： 190.00 到 600.00
扫描速度： 中速
采样间隔： 0.5
自动采样间隔： 停用
扫描模式： 单个

[仪器属性]
仪器类型： UV-2700 系列
测定方式： 吸收值
狭缝宽： 5.0 nm
积分时间： 0.1 秒
光源转换波长： 323.0 nm
检测器单元： 直接
S/R 转换： 标准
阶梯校正： OFF

[附件属性]
附件： 无

[数据处理参数]
阈值： 0.0010000
点： 4
内插： 停用
平均： 停用

[样品准备属性]
重量：
体积：
稀释：
光程长： 10mm
附加信息： 样品浓度：0.0432毫克/毫升
溶剂：甲醇

Wavelength Range:190-600
Scan Speed: Medium
Sampling Interval:0.5
Auto Sampling Interval: Enabled
Scan Mode: Auto

Instrument Properties
Instrument Type: UV-2700PC Series
Measuring Mode: Absorbance
Slit Width: 5.0 nm
Light Source Change Wavelength: 325.0 nm
S/R Exchange: Normal

Sample concentration: 0.0432 mg/mL
Solvent: MeOH

No.	P/V	波长(nm)	Abs.	描述
1	↑	273.50	0.045	
2	↑	196.50	0.505	
3	↓	259.50	0.040	

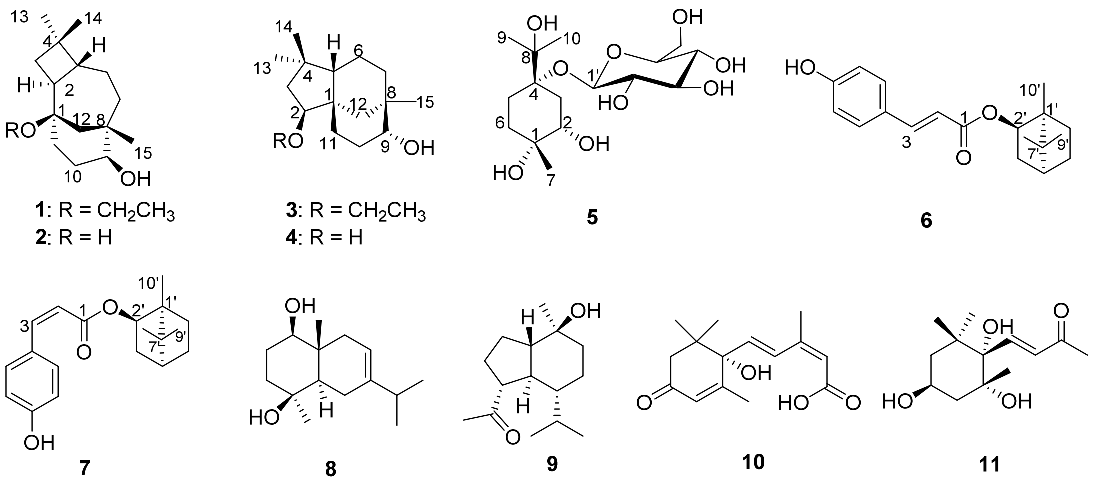

Fig.1. The structure of compounds **1-11**

Caryolane-1, 9β-diol (2): White amorphous powder; ^{1}H-NMR (400 MHz, CDCl3) δ: 3.43 (1H, t, *J* = 4.0 Hz, H-9), 2.22 (1H, m, H-2), 2.04 (1H, m, H-10a), 1.88 (1H, m, H-5), 1.76 (1H, m, H-10b), 1.63 (1H, m, H-11a), 1.55 (1H, m, H-3a), 1.52 (1H, m, H-6a), 1.50 (1H, m, H-11b), 1.45 (1H, m, H-3b), 1.40 (3H, m, H-12, H-7a), 1.38 (1H, H-6b), 1.15 (1H, m, H-7b), 1.01 (3H, s, H-14), 1.00 (3H, s, H-13), 0.92 (3H. s, H-15); ^{13}C-NMR (100 MHz, CDCl3) δ: 70.7 (s, C-1), 38.0 (d, C-2), 34.0 (t, C-3), 35.1 (s, C-4), 43.9 (d, C-5), 20.4 (t, C-6), 35.4 (t, C-7), 39.3 (s, C-8), 72.3 (d, C-9), 28.2 (t, C-10), 33.4 (t, C-11), 42.4 (t, C-12), 20.8 (q, C-13), 30.5 (q, C-14), 26.6 (q, C-15); EI-MS *m/z*: 337 ([M-H]$^{+}$, 5), 286 (10), 150 (100), 149 (50), 69 (100)。

Clovane-2α,9β-diol (4): White amorphous powder; ^{1}H-NMR (400 MHz, CDCl3) δ: 3.80 (1H, dd, *J* = 5.6, 10.4 Hz, H-2), 3.33 (1H, br s, H-9), 1.99 (1H, m, H-10a), 1.72 (2H, m, H-11a, H-3a), 1.66 (1H, m, H-10b), 1.55 (1H, m, H-12a), 1.42 (3H, m, H-6a, H-7a, H-5), 1.31 (1H, m, H-6b), 1.12 (1H, m, H-11b), 1.09 (1H, m, H-3b), 1.04 (3H, s, H-14), 0.97 (3H, s, H-15), 0.93 (1H, m, H-12b), 0.86 (3H, s, H-13); ^{13}C-NMR (100 MHz, CDCl3) δ: 44.1 (s, C-1), 80.8 (d, C-2), 47.3 (t, C-3), 37.1 (s, C-4), 50.5 (d, C-5), 20.6 (t, C-6), 33.0 (t, C-7), 34.7 (s,

C-8), 75.2 (d, C-9), 26.3 (t, C-10), 26.7 (t, C-11), 35.5 (t, C-12), 25.4 (q, C-13), 31.4 (q, C-14), 28.4 (q, C-15)。

(-)-Bornyl p-coumarate (**6**): White amorphous powder; ^{1}H-NMR (600 MHz, CD_3OD) δ: 7.62 (1H, d, *J* = 15.9 Hz, H-2), 7.49 (2H, d, *J* = 8.6 Hz, H-5, H-9), 6.82 (2H, d, *J* = 8.6 Hz, H-6, H-8), 6.36 (1H, d, *J* = 15.9 Hz, H-3), 5.01 (1H, m H-2'), 2.43 (1H, m, H-3'b), 2.11 (1H, m, H-6'b), 1.84 (1H, m, H-5'b), 1.72 (1H, t, *J* = 4.5 Hz, H-4'), 1.42 (1H, m, H-6'a), 1.33 (1H, m, H-5'a), 1.06 (1H, dd, *J* = 13.7, 3.6 Hz, H-3'a), 0.99 (3H, s, 9'-CH_3), 0.95 (3H, s, 8'-CH_3), 0.91 (3H, s, 10'-CH_3);^{13}C-NMR (150 MHz, CD_3OD) δ: 168.3 (s, C-1), 159.9 (s, C-7), 144.9 (d, C-3), 129.8 (d, C-5, C-9), 125.8 (s, C-4), 115.4 (d, C-2), 114.1 (d, C-6, C-8), 79.7 (d, C-2'), 48.5 (s, C-1'), 47.5 (s, C-7'), 44.9 (d, C-4'), 36.5 (t, C-3'), 27.6 (t, C-5'), 26.8 (t, C-6'), 18.7 (q, C-8'), 17.8 (q, C-9'), 12.5 (q, C-10')。

(-)-Bornyl cis-4-hydroxycinnamate **(7):** White amorphous powder; ^{1}H-NMR (600 MHz, CD_3OD) δ: 7.58 (2H, d, *J* = 8.6 Hz, H-5, H-9), 6.89 (1H, d, *J* = 12.7 Hz, H-2), 6.76 (2H, d, *J* = 8.7 Hz, H-6, H-8), 5.81 (1H, d, *J* = 12.7 Hz, H-3), 4.94 (1H, m, H-2'), 2.41 (1H, m, H-3'b), 1.86 (1H, m, H-6'b), 1.77 (1H, m, H-5'b), 1.69 (1H, t, *J* = 4.5 Hz, H-4'), 1.04 (1H, dd, *J* =13.7, 3.6 Hz, H-3'a), 1.29 (1H, m, H-6'a), 1.22 (1H, m, H-5'a), 0.96 (3H, s, 9'-CH_3), 0.92 (3H, s, 8'-CH_3), 0.82 (3H, s, 10'-CH_3);^{13}C-NMR (150 MHz, CD_3OD) δ: 167.6 (s, C-1), 158.5 (s, C-7), 143.0 (d, C-3), 131.9 (d, C-5, C-9), 126.4 (s, C-4), 116.1 (d, C-2), 114.5 (d, C-6, C-8), 79.8 (d, C-2'), 48.3 (s, C-1'), 47.6 (s, C-7'), 44.9 (d, C-4'), 36.3 (t, C-3'), 27.5 (t, C-5'), 26.7 (t, C-6'), 18.7 (q, C-8'), 17.8 (q, C-9'),12.5 (q, C-10')。

Oplodiol (8): White amorphous powder; ^{1}H-NMR (500 MHz, $CDCl_3$) δ: 5.63 (1H, d, *J* = 5.0 Hz, H-8), 3.32 (1H, dd, *J* = 11.5, 4.0 Hz, H-1), 2.22 (1H, m, H-11), 2.09 (1H, m, H-2a), 2.06 (2H, m, H-6), 1.89 (1H, m, H-9a), 1.86 (1H, m, H-2b), 1.76 (1H, m, H-3a), 1.63 (1H, m, H-9b), 1.59 (1H, m, H-3b), 1.32 (1H, m, H-5), 1.20 (3H, s, H-15), 1.05 (3H, d, *J* = 6.5 Hz, H-12), 1.04 (3H, d, *J* = 6.5 Hz, H-13), 0.98 (3H, s, H-14); ^{13}C-NMR (125 MHz, $CDCl_3$) δ: 79.7 (d, C-1), 40.7 (t, C-2), 39.4 (t, C-3), 71.0 (s, C-4), 46.2 (d, C-5), 23.0 (t, C-6), 141.9 (s, C-7), 116.1 (d, C-8), 26.9 (t, C-9), 37.7 (s, C-10), 35.0 (d, C-11), 21.8 (q, C-12), 21.2 (q, C-13), 11.7 (q, C-14), 19.8 (q, C-15)。

Ent-oplopanone (9): White amorphous powder; ^{1}H-NMR (400 MHz, $CDCl_3$) δ: 2.64 (1H, m, H-4), 2.19 (3H, s, H-14), 1.20 (3H, s,

H-15), 0.89 (3H, d, J = 6.7 Hz, H-11), 0.68 (3H, d, J = 6.7 Hz, H-12); ^{13}C-NMR (100 MHz, $CDCl_3$) δ: 46.7 (d, C-1), 42.0 (t, C-2), 28.6 (t, C-3), 57.0 (d, C-4), 211.5 (s, C-5), 55.7 (d, C-6), 49.4 (d, C-7), 25.3 (t, C-8), 23.0 (t, C-9), 73.0 (s, C-10), 29.5 (d, C-11), 15.6 (q, C-12), 22.0 (q, C-13), 20.3 (q, C-14), 29.5 (q, C-15)。

Abscisic acid (10): White amorphous powder; ^{1}H-NMR (600 MHz, CD_3OD) δ: 7.77 (1H, d, J = 16.1 Hz, H-4), 6.24 (1H, d, J = 16.1 Hz, H-5), 5.92 (1H, s, H-3′), 5.74 (1H, s, H-2), 2.53 (1H, d, J = 17.0 Hz, H-5′a), 2.18 (1H, d, J = 17.0, H-5′b), 2.04 (3H, s, H-6), 1.93 (3H, s, H-7′), 1.06 (3H, s, H-8′), 1.03 (3H, s, H-9′); ^{13}C-NMR (150 MHz, CD_3OD) δ:168.0 (s, C-1), 126.2 (d, C-2), 149.7 (s, C-3), 128.0 (d, C-4), 136.6 (d, C-5), 20.0 (q, C-6),79.2 (s, C-1′),165.1 (s, C-2′), 118.2 (d, C-3′), 199.8 (s, C-4′), 49.3 (t, C-5′), 41.5 (s, C-6′), 18.3 (q, C-7′), 22.2 (q, C-8′), 23.3 (q, C-9′).

(3S,5R,6S,7E)-3,5,6-trihydroxy-7-megastigmen-9-one (11): White amorphous powder; ^{1}H-NMR (400 MHz, CD_3OD) δ:7.17 (1H, d, J = 15.8 Hz, H-8), 6.17 (1H, d, J = 15.7 Hz, H-8), 3.76 (1H, m, H-3), 2.40-2.57 (1H, m, H-4a), 2.29 (3H, s, H-10), 1.64-1.68 (1H, m, H-4b), 1.55-1.59 (1H, m, H-2a), 1.23-1.27 (1H, m, H-2b), 1.19 (3H, s, H-12), 1.18 (3H, s, H-13), 0.95 (3H, s, H-11); ^{13}C-NMR (100 MHz, $CDCl_3$) δ:^{13}C-NMR (100 MHz, CD_3OD) δ: 34.7 (s, C-1), 46.3 (t, C-2), 63.0 (d, C-3), 39.9 (t, C-4), 67.4 (s, C-5), 69.5 (d, C-6), 144.0 (d, C-7), 132.4 (d, C-8), 198.8 (s, C-9), 26.0 (q, C-10), 23.8 (q, C-11), 128.4 (q, C-12), 8.7 (q, C-13).

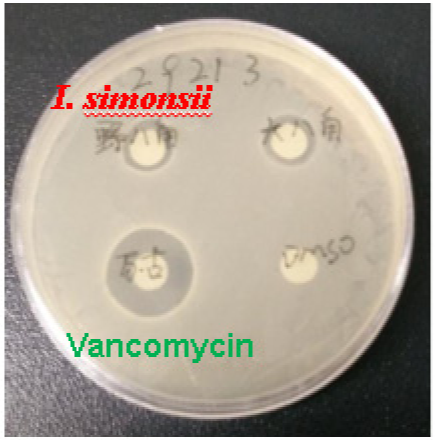

S. aureus ATCC29213

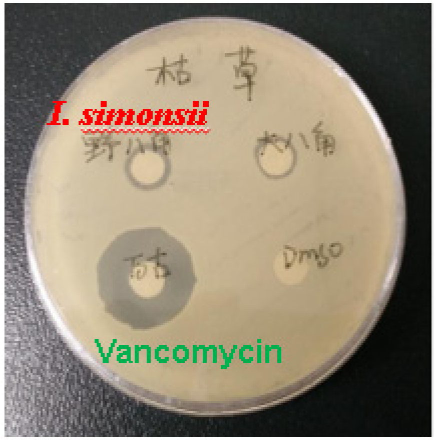

B. subtilis ATCC6633

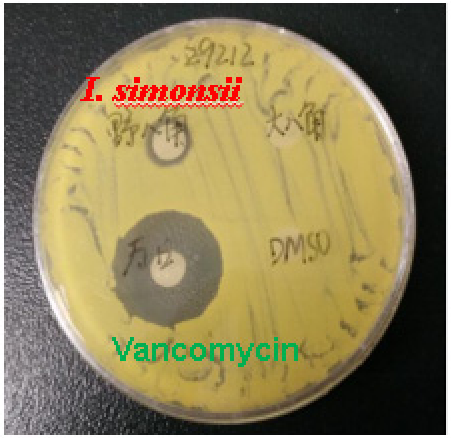

E. faecalis ATCC29212
